# A neuroradiologist’s guide to arterial spin labeling MRI in clinical practice

**DOI:** 10.1007/s00234-015-1571-z

**Published:** 2015-09-09

**Authors:** M. Grade, J. A. Hernandez Tamames, F. B. Pizzini, E. Achten, X. Golay, M. Smits

**Affiliations:** UCL Institute of Neurology, Queen Square, London, UK; Stanford School of Medicine, Stanford, CA USA; Medical Image Analysis and Biometry Laboratory, Rey Juan Carlos University, Móstoles, Madrid, Spain; Neuroradiology, Department of Diagnostics and Pathology, Verona University Hospital, Verona, Italy; Neuroradiology, Department of Radiology, Ghent University Hospital, Ghent, Belgium; Department of Radiology, Erasmus MC—University Medical Centre Rotterdam, PO Box 2040, 3000 CA Rotterdam, The Netherlands

**Keywords:** Arterial spin labeling, Perfusion, Dementia, Brain tumour, Stroke

## Abstract

Arterial spin labeling (ASL) is a non-invasive MRI technique to measure cerebral blood flow (CBF). This review provides a practical guide and overview of the clinical applications of ASL of the brain, as well its potential pitfalls. The technical and physiological background is also addressed. At present, main areas of interest are cerebrovascular disease, dementia and neuro-oncology. In cerebrovascular disease, ASL is of particular interest owing to its quantitative nature and its capability to determine cerebral arterial territories. In acute stroke, the source of the collateral blood supply in the penumbra may be visualised. In chronic cerebrovascular disease, the extent and severity of compromised cerebral perfusion can be visualised, which may be used to guide therapeutic or preventative intervention. ASL has potential for the detection and follow-up of arteriovenous malformations. In the workup of dementia patients, ASL is proposed as a diagnostic alternative to PET. It can easily be added to the routinely performed structural MRI examination. In patients with established Alzheimer’s disease and frontotemporal dementia, hypoperfusion patterns are seen that are similar to hypometabolism patterns seen with PET. Studies on ASL in brain tumour imaging indicate a high correlation between areas of increased CBF as measured with ASL and increased cerebral blood volume as measured with dynamic susceptibility contrast-enhanced perfusion imaging. Major advantages of ASL for brain tumour imaging are the fact that CBF measurements are not influenced by breakdown of the blood–brain barrier, as well as its quantitative nature, facilitating multicentre and longitudinal studies.

## Introduction

The measurement of perfusion has become an indispensable tool in the clinical evaluation of the brain. A number of methodologies can be applied for this purpose, each with its own advantages and disadvantages [1]. While dynamic susceptibility contrast (DSC)-magnetic resonance imaging (MRI), computed tomography (CT) perfusion imaging, single-photon emission tomography (SPECT), and H2[15O] positron-emission tomography (PET) are well-established methods for investigating blood flow in neurological diseases, arterial spin labeling (ASL) MRI has emerged as a versatile complement that warrants regular consideration in the clinical setting.

SPECT, CT, PET, and DSC-MRI measure perfusion by dynamic imaging of the passage of a contrast agent. By contrast, ASL generates an image by magnetically “labeling” water molecules as an endogenous tracer as they travel to an organ of interest. Selective radiofrequency (RF) irradiation inverts the magnetisation of arterial blood water in the region or plane to which it is applied, usually in the neck for brain perfusion, and a downstream measurement is taken as labeled spins exchange into the tissue of interest [2]. In most ASL methods, the resulting images are compared to control images in which no inversion pulse is applied. The difference reveals the perfusion, indirectly related to the quantification of cerebral blood flow (CBF) in well-characterised units of millilitres of blood per 100 g of tissue per minute.

The physical basis of ASL offers its greatest advantage over traditional contrast bolus techniques: it is non-invasive. ASL does not require a gadolinium-based tracer, eliminating risk of nephrogenic systemic fibrosis in patients with renal dysfunction [3]. ASL is also favourable for paediatric populations, as it avoids the technical difficulties and ethical problems of contrast agents and radiation exposure with CT and nuclear medicine techniques [4]. In addition, this makes the method easily repeatable, a trait that is useful for performing perfusion-based functional MRI (fMRI) and evaluating changes over time [5]. Reproducibility has been addressed in a number of studies, verifying the potential of ASL for longitudinal monitoring of CBF changes [6–9].

The main drawback of ASL is the signal-to-noise ratio (SNR), which is inherently low because inflowing labeled molecules comprise only about 1 % of the static tissue signal [10]. This increases the total necessary scan time, making the technique particularly sensitive to motion artefact [11]. Flow quantification can be complex, as the signal is dependent on a number of physiological parameters. Sources of error include transit time, inversion pulse profile, and labeled blood that does not perfuse [12].

Publications about ASL in neurological diseases have increased in recent years. Technological improvements in MRI scanners and ASL methods are undeniable, and the popularity of ASL in clinically relevant research is growing as non-specialists become acquainted with commercially available acquisition and analysis software. However, routine clinical use of ASL is still limited to few centres [13]. Detre et al. [14] find it ‘perplexing’ that the method has not been incorporated into routine clinical practice, while Golay and Guenther [13] perceive a ‘lack of enthusiasm from the clinical community’. Why is this? There are a number of potential factors, such as the low SNR compared to other imaging modalities. ASL techniques can be complicated and have traditionally not been widely compatible with commercial scanners. The utility and benefits of ASL are often eclipsed by the greater prevalence of modalities such as DSC-MRI; some clinicians may not request ASL as they are not accustomed to non-invasive CBF quantification [14]. In addition to a lack of awareness of the potential clinical utility of ASL, several other issues can be identified that seem to impede widespread clinical use. These include difficulties with image post-processing, a wide variety of available acquisition techniques and parameters, and a lack of guidelines for interpretation [15]. Communication may resolve some of these issues. The establishment of the ASL network has been a joint effort to rectify the ASL information gap by providing a centralised communication platform [13]. The recently published white paper on the clinical implementation of ASL [16] aims to reduce the confusion that comes with the many different implementations and provides clear guidelines for sequence implementation.

In this review paper, we describe how to use ASL imaging of the brain clinically, particularly for those neurological diseases with ample evidence of ASL’s clinical value: cerebrovascular disease, dementia, and neuro-oncology. We also provide some technical background and recommendations on how to optimally acquire ASL images in line with the white paper [16]. We aim to provide the reader with clinically relevant and practical information to confidently implement and use ASL of the brain in their routine practice.

## ASL technique and acquisition

ASL provides quantitative parametric images of tissue perfusion. For that purpose, it uses the water in arterial blood as an endogenous, freely diffusible contrast medium. The main physiological parameter that is measured with ASL is CBF, which determines the delivery rate of oxygen and nutrients to the capillary bed and is expressed as the volume of blood per volume of tissue per minute (ml 100 g^−1^ min^−1^).

### Physical and physiological principles

**Fig. 1:**
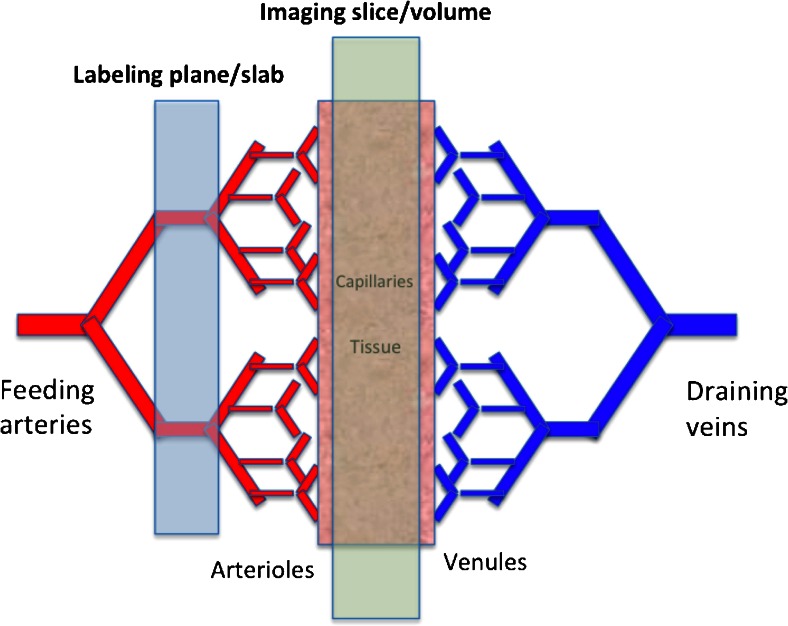
Capillary bed where water and nutrient exchange with the brain parenchyma takes place

**Fig. 2:**
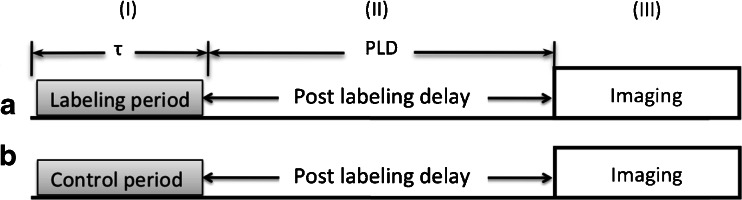
Schematic of the three periods in any ASL sequence: (*I*) labeling/control period, (*II*) post-labeling delay (PLD), to allow for the magnetisation to reach and exchange with the tissue, and (*III*)) imaging period, during which the images of the brain are acquired. **a** Labeling acquisition. **b** Control acquisition. The difference between the two acquisitions only lies in the type of radiofrequency pulse and gradient applied during the first part of the sequence (yes or no labeling)

ASL’s aim is the assessment of tissue perfusion rate, which is very different from macrovascular blood flow. Tissue perfusion, or the exchange of water and nutrients with the tissue, happens along the entire length of the capillaries (Fig. 1). ASL basically ‘follows’ blood water molecules from the arterial compartment all the way to the tissue capillary bed, using them as a free diffusible tracer. ASL is easily carried out by the inversion or saturation of the magnetisation along the *Z*-axis of blood water molecules in the feeding arteries. This part of the ASL acquisition is called the labeling. Following the labeling, time is allowed for the blood to travel to the tissue: the so-called post-labeling delay (PLD) or inversion time for certain ASL techniques. The delay is chosen such that images are ideally acquired at the time of exchange of the water molecules with the tissue magnetisation (Fig. 2). Arterial blood labeling is achieved by a combination of RF pulses and gradients in order to invert the longitudinal magnetisation (T1) of blood water protons.

After the PLD, the image acquisition phase starts to obtain signal from those labeled protons coming from the feeding arteries into the tissue. Different types of readout can be employed. In order to differentiate the labeled magnetisation signal from the static tissue magnetisation signal, two images are acquired: one with (labeled image, Fig. 2a) and one without (control image, Fig. 2b) labeling. Subtraction of the labeled from the control images provides the perfusion-weighted image.

Most ASL techniques aim to avoid signal coming either from the flow in vessels or from static tissue, employing for that purpose additional gradient and RF pulses. In addition, numerous pulse sequences have been developed to maximise SNR by reducing all confounding and potentially artefactual signals. In particular, multiple cycles of control and labeled images are acquired, leading to typically 4–6-min acquisition times. The need to acquire multiple repetitions may however lead to additional artefacts, such as those due to patient motion, and necessitate the use of additional strategies to obtain usable ASL data either during acquisition or with post-processing techniques.

Several physical and physiological parameters will affect the quality of the ASL image and need to be understood prior to choosing the most appropriate sequence and parameters. Relevant factors include the labeling efficiency, arterial blood T1 and relaxation times, the blood transport time through vessels and tissue (depending on flow velocity), and finally the so-called magnetisation transfer effects.

Arterial labeling efficiency is the first key aspect to a good ASL acquisition. How effectively we invert the longitudinal magnetisation directly affects the eventually acquired signal. Efficiencies, designated with *α*, can range from 80 to 98 % with currently available pulse sequences, which need to be taken into account when quantifying CBF [2, 17–20].

Both blood transit time and T1 relaxation are in the order of seconds, meaning that about two thirds of the label will have decayed by the time the blood reaches the capillary bed. The optimal PLD is thus almost invariably a compromise between the arterial blood T1 and transit time. Arterial T1 and feeding arteries transit times both need to be taken into account to get a proper absolute quantification by physiological modelling [21]. Ideally, the arterial blood relaxation time (T1_Blood_) is measured on an individual basis. In the capillary bed, the arterial blood water will exchange with the surrounding tissue. The ratio of tissue water versus intravascular water in the brain is in the order of 20:1, meaning that only a very small proportion of the measured water signal is affected by the magnetic label. When the labeled water enters the tissue, over 90–95 % exchanges with the tissue water instantaneously. For this reason, it is impossible to measure any labeled venous blood flowing out of the tissue, due to the very short T1 relaxation time compared with the equilibrium time (1–2 s versus 10–15 min for a nominal CBF of 50 ml/min/100 g, respectively) [22].

One of the major confounds when using ASL comes from magnetisation transfer effects. The application of off-resonance RF pulses to label the arterial blood proximal to the tissue of interest will induce a reduction of MR signal that mimics the signal decrease induced by the labeled blood water exchange. This is due to the fact that protons in macromolecules have a very short T2 and thus a very broad frequency spectrum. As such, the off-resonance RF pulses used in any asymmetrical ASL sequence will inadvertently not only saturate the arterial blood but also protons in these macromolecules. This saturation will then be transferred to tissue water molecules reducing the MR signal [23].

Three main categories of ASL pulse sequences have been developed, each aiming to take these factors into account with variable success: continuous ASL (CASL), pseudocontinuous ASL (pCASL), and pulsed ASL (PASL). PCASL is at present the sequence of choice, due to its high labeling efficiency combined with its ease of implementation and hardware specifications for clinical scanners [16].

### The ASL pulse sequence

#### Labeling methods

**Fig. 3:**
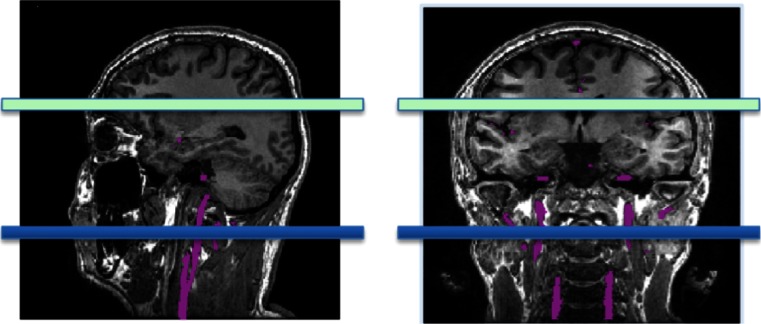
Labeling slice prescription (in *blue*) in sagittal and coronal views for (p)CASL. Feeding arteries are shown in *purple*

The first labeling method was CASL and was introduced in 1992 [2, 24]. The simultaneous application of both a constant RF pulse and gradient G_z_ prescribes a well-defined labeling plane where inflowing blood is continuously labeled during 2–4 s (labeling duration) (Figs. 2 and 3). Image acquisition occurs downstream from the labeled blood, usually with a large field of view covering the entire brain, after the PLD. CASL, however, is strongly affected by magnetisation transfer effects due to the long RF pulse. Additionally, due to the continuous application of a low amplitude RF labeling pulse, this sequence is usually difficult to implement on clinical scanners.

PCASL deals with some of CASL’s disadvantages. In particular, pCASL avoids the need of additional hardware. Also, specific absorption rate (SAR) deposition is lower than in CASL. PCASL uses series of short RF and gradient pulses instead of the long RF and gradient pulses employed in CASL. This train of short pulses results in an inversion with the same effect and efficiency as with CASL. One drawback in pCASL is its sensitivity to resonance offset at the labeling plane, which can cause it to shift. Additionally, some spin phase shift between RF pulses can occur. Both effects can potentially reduce the labeling efficiency. Such effects can be however minimised by reducing the time gap between the consecutive RF pulses or through post-processing.

**Fig. 4:**
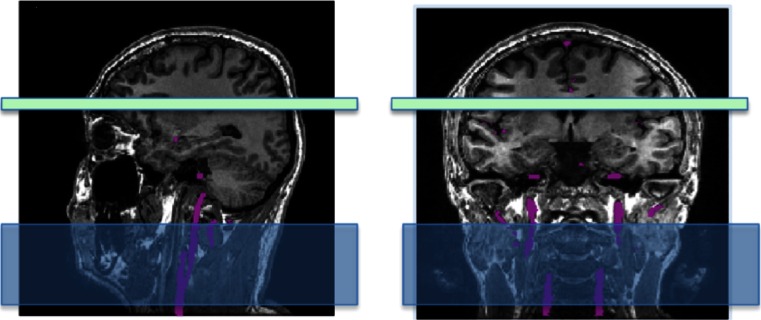
Labeling slice prescription (in *blue*) in sagittal and coronal views for PASL. Feeding arteries are shown in *purple*

In contrast to (p)CASL, in PASL a large slab is inverted along feeding arteries during the very brief labeling phase (Fig. 4). The labeled bolus size thus directly depends on the slab size and not on the labeling duration such as in (p)CASL. There are many different implementations of pulsed ASL sequences, each applying different strategies [25]. With PASL, the labeling efficiency is high while SAR is lower than in CASL or pCASL. This, however, comes at the cost of a lower SNR, which is theoretically only 70 % of the maximal SNR achievable using (p)CASL [21, 26–28].

#### Background suppression

The signal from static tissue fluctuates due to physiological and thermal noise effects. This interacts with the signal derived from ASL, thereby increasing the physiological noise in the image. Therefore, after the labeling duration (Fig. 2), additional RF pulses are applied to reduce the tissue signal [29].

#### Readout

The traditional readout scheme for ASL makes use of fast imaging techniques. Until recently, echo planar imaging (EPI) has been the preferred acquisition technique. However, more advanced methods now provide alternative readouts, such as 3D-gradient and spin echo (GRASE) [30]. This sequence has great advantages for ASL because it allows acquisition of the entire volume of interest in a single shot, thereby reducing the slice-dependent variation in perfusion signal due to differences in acquisition delays that are inherent to 2D multi-slice methods. 3D GRASE also has superior SNR compared to 2D acquisitions. An alternative to 3D GRASE is 3D rapid acquisition relaxation enhanced (RARE) combined with a spiral readout trajectory [31]. This sequence oversamples the centre of the k-space, thus providing shorter echo times and superior SNR, but can introduce through-plane blurring due to long readout times [32]. Such blurring can be reduced by segmenting the acquisition of the 3D readout into multiple acquisitions.

### How to acquire your ASL scan

The white paper recommends a pCASL labeling scheme, combined with background suppression and a segmented 3D readout. SNR will be better at high field, so 3 T is preferred, especially in patient populations with slow or poor blood flow [16]. At 1.5 T, ASL is feasible when blood flow is not too compromised and in the paediatric population but will require longer scanning times to obtain sufficient signal.

Phased array coils (eight or more channels) are recommended to provide higher SNR especially in the cortex. These coils also allow parallel imaging techniques and may thereby accelerate the acquisition [33].

#### Field strength

ASL is a low SNR technique and as such benefits from any increase in SNR possible. As the signal in MRI grows quadratically with the field strength, while the noise only linearly, the SNR is expected to grow theoretically linearly with the field strength [34]. In addition to a general higher SNR, higher field strengths provide longer blood T1 relaxation, leading to a further increase in SNR, especially in cases of slow flow [35, 36]. The potential of ultrahigh field strength imaging (i.e., at 7 T or higher) is discussed below under ‘Emerging applications and techniques’.

ASL at high field, however, also comes with disadvantages. First, not only T1 but also T2 will be affected by field strength and will generally be much shorter at 3.0 than 1.5 T [34], with a concomitant reduction in T2*. This will result in a slight reduction of SNR due to the long readouts usually employed in ASL (based on EPI or a combination of EPI with multiple spin echoes such as 3D GRASE), during which some of the available signal from the exchanged labeled blood will have disappeared. This effect should be accounted for in the quantification if one does not want to underestimate perfusion at higher field strength [37]. Second, both the applied and main magnetic fields will in principle be less homogenous at 3.0 than 1.5 T. Finally, the move to higher field strengths comes with an increase in the RF deposition. Advanced methods including optimised RF shimming have now been developed to reduce hot spots in the applied RF field and are available for all ASL protocols.

**Fig. 5:**
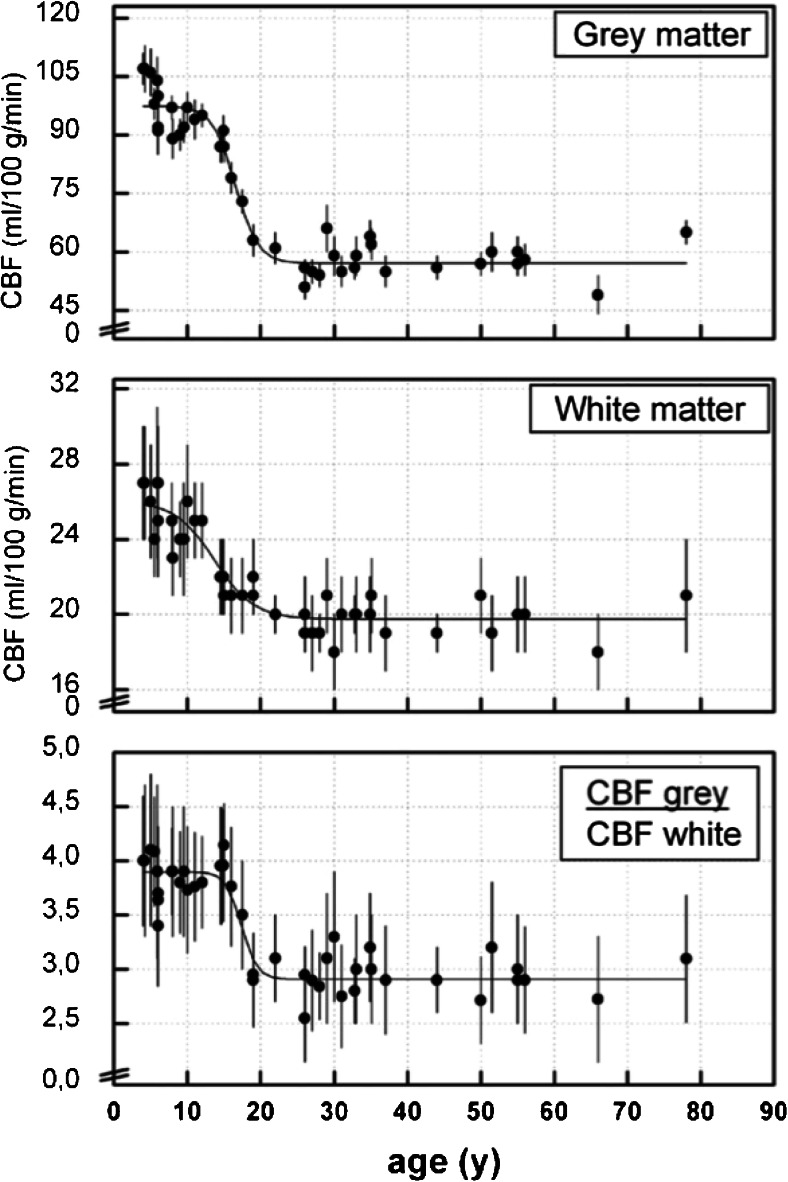
Cerebral blood flow at different ages, for grey matter, white matter, and their ratio. Reprinted with permission from [37]

All in all, despite these disadvantages, 3.0 T has been shown to be much better suited for ASL than 1.5 T, as the increase in SNR overcomes most of the potential side effects of high field strength scanners. This does not mean that 1.5 T cannot be used and can in fact be ideal for certain applications where a relatively high perfusion is expected, such as in children between 2 and 18 years old [38] (Fig. 5), or in other organs, such as the kidneys, for which the advantages of lower field (i.e., reduced main and applied magnetic field inhomogeneities and reduced RF deposition) will counterbalance the loss of SNR.

#### Acquisition parameters

For pCASL, the most important acquisition parameters to choose are the labeling duration and the PLD.

The choice of PLD is highly dependent on the—presumed—blood velocity. The recommended PLD is 2,000 ms in neonates, 1,500 ms in children, 1,800 ms in healthy adults <70 years of age, and 2,000 ms in healthy adult >70 year of age. In adult patients, 2,000 ms is generally recommended, to allow sufficient time for proper delivery of the blood to the tissue.

For spatial resolution, 3–4-mm in-plane and 4–8-mm through-plane are recommended to achieve sufficient SNR. In segmented acquisitions such as 3D RARE stack-of-spiral or 3D GRASE, it is recommended to use 8 to 12 segments or arms. If the readout is 2D EPI or spiral single shot, a minimum echo time is desirable. Scan time for clinical purposes should take no longer than 4–5 min, to minimise artefacts related to motion. Further recommendations, also for PASL and CASL, can be found in Alsop et al. [16].

#### Positioning

**Fig. 6:**
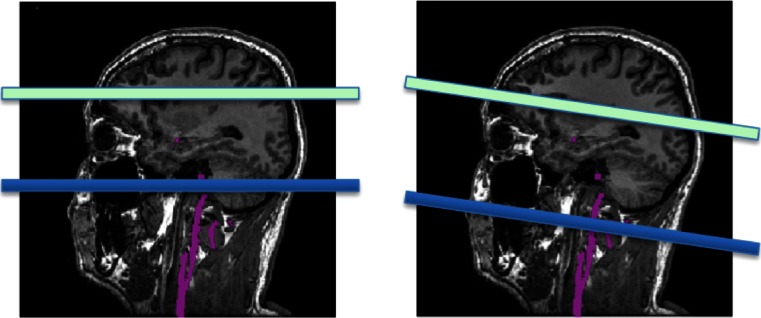
Incorrect (*left*) and correct (*right*) positioning of the labeling plane. The labeling plane needs to be placed perpendicular to the feeding arteries and sources of susceptibility artefact (such as air in the sinuses) should be avoided

The labeling plane must be located in a region where the relevant feeding arteries are relatively straight and perpendicular to the labeling plane. Figure 6 shows examples of both bad and good positioning of the labeling plane. Prior to positioning, an MR angiogram may be acquired such that the feeding vessels are visualised. A quick, low-resolution angiogram is sufficient for this purpose. Alternatively, other landmarks can be used. The labeling plane can be placed approximately 8–9 cm inferior to the anterior–posterior commissure line in adults or approximately 1 cm below the inferior border of the cerebellum. A caveat with positioning the labeling plane like this is that the lower cerebellar sections may suffer from artefacts due to direct saturation from the RF pulses.

**Fig. 7:**
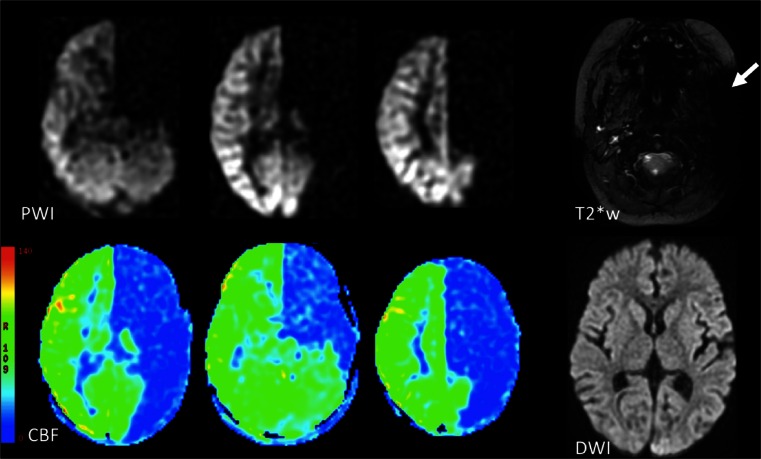
Labeling failure in the left internal carotid artery. There is absence of signal on the ASL perfusion-weighted images (*PWI*) and corresponding cerebral blood flow (*CBF*) maps in the arterial territory of the left internal carotid artery. Note the large susceptibility artefact in the area of the left internal carotid area on the T2* weighted (T2*w) image, as the likely cause of the labeling failure. Diffusion weighted imaging (*DWI*) is normal, supporting interpretation of this finding as an artefact

It is extremely important that the labeling plane does not include any sources of susceptibility effects such as air or bone close to the orbitofrontal or temporal areas but also dental fillings or metallic surgical material. These sources of susceptibility can ruin the labeling and may result in a lack of signal in the vascular territory of the vessel in which labeling failed (Fig. 7).

#### Motion

Due to the fact that perfusion-weighted imaging with ASL is a subtraction technique, combined with the necessity of many ‘control-label’ repetitions, ASL is highly sensitive to motion. Background suppression substantially reduces motion artefacts. Remaining motion artefacts can be further reduced by using image registration post-processing techniques similar to those used in functional MRI.

## Clinical applications

Perfusion patterns in various neurological diseases differ because they reflect different mechanisms. Although it is useful to group incidences of hyperperfusion and hypoperfusion in large-scale analysis of ASL findings [39, 40], one needs to keep in mind that such outcomes can be indicative of a number of pathologies. ASL depicts physiology. Stroke and ischaemia yield hypoperfusion because they result from disruptions of brain haemodynamics. In contrast, regional hypoperfusion patterns in neurodegenerative diseases likely reflect a progressive breakdown of synaptic pathways. Hyperperfusion in brain tumours is a result of greater metabolic needs of uncontrolled cell replication, whereas ictal hyperperfusion recruits metabolites for uncontrolled electrical discharges.

### Cerebrovascular disease

The measurement of perfusion is probably most directly applicable to cerebrovascular disease and ischaemic episodes. These conditions typically manifest themselves on ASL as regions of altered perfusion or delayed arterial transit time (ATT) because malformed, occluded, and damaged vessels perfuse less labeled water than normal vessels [41]. ASL has been explored for the non-invasive detection of cerebrovascular abnormalities, identification of ischemic events, and risk assessment. The technique can be combined with an acetazolamide challenge to assess the cerebrovascular reserve [42, 43]. The development of selective ASL, which can visualise the vascular territory of a single vessel, is particularly applicable to these investigations [44].

The physiological basis of ASL may however pose difficulties in patients with suspected cerebrovascular disease. Qualitative assessment of cerebral blood flow maps can be clinically useful but may be insufficient and inaccurate in the presence of globally reduced cerebral blood flow, such as in patients with bilateral carotid disease or reduced cardiac output. ATT is well-defined for healthy vasculature but can vary greatly in disease states, possibly making conventional ASL techniques with a single PLD unsuitable [45]. Slow flow is more difficult to quantify than high flow [46]. Observed variability in ATT for chronic stroke patients, for example, indicates that individual transit delay times ought to be established in cases of compromised cerebral vasculature before collecting CBF data [47]. Recent work demonstrated a multi-PLD technique that measures both CBF and ATT, creating parametric maps of regional ATT to better model pathological tissue [48, 49]. The technique was recently validated against gold standard H2[15O]-PET measurements [43] and DSC [50] in instances of steno-occlusive disease.

#### Vascular disease

ASL is useful for identifying abnormal cerebral perfusion caused by stenosis or constriction of blood vessels. ICA occlusion [51, 52] and stenosis [53] have been identified with ASL by delayed ATT in the hemisphere ipsilateral to the affected vessel, findings that are validated by correlation with SPECT [54]. In addition, surgical correction by carotid artery stenting has been quantified with pCASL [55]. In Moyamoya disease (MMD), the extent of collateral perfusion, which is one of the surrogate markers of disease severity and stroke risk, can be assessed with ASL. Measurements reportedly agree with those of gold standard techniques [56]. Most recently, Donahue et al. [57] correlated ASL and BOLD measurements of haemodynamics with digital subtraction angiography (DSA) arterial circulation time, an established marker of disease severity, determining that the non-invasive MRI methods provide complementary tissue-level information about parenchymal reserves. ASL measurements of CBF for MMD have been correlated with SPECT [58], as well as H2[15O]-PET in young adults [59]. Clinical trials evaluating the predictive value of ASL for identifying high-risk MMD patients are warranted. Early identification of at-risk brain tissue in cases of neurotuberculosis [60] and Hashimoto’s encephalopathy [61]—the latter a case study in which only CASL detected abnormal hyperperfusion—support the inclusion of ASL in investigative imaging protocols.

Arteriovenous malformation (AVM) has an associated ‘steal phenomenon’ that has been visualised with PASL [62]. PASL was also shown to be useful in measuring the amount of shunt reduction achieved after embolisation of AVMs [63], as well as confirming obliteration of AVM after stereotactic radiosurgery [64, 65]. Vessel-encoded pCASL was found to be as good as DSA at identifying AVM feeding vessels and their contribution fractions [66].

In addition to verifying surgery outcomes, the ease of repeat ASL measurements of time-dependent CBF changes could be useful in post-treatment monitoring. Serial ASL can track the status of patients at risk of angiographic vasospasm in the days following subarachnoid haemorrhage [67]. Selective ASL is useful in monitoring haemodynamic changes and potential risk of cerebral hyperperfusion after revascularisation surgery for MMD [68, 69]. Similarly, serial PASL upon insertion of transjugular intrahepatic portosystemic shunts in cirrhotic patients could help predict hepatic encephalopathy [70].

#### (Sub)acute ischaemia

Ischaemia is identifiable by regions of altered perfusion due to anoxic damage. ASL has been used to measure global hypoperfusion in individuals in a minimally conscious state due to traumatic brain injury, stroke, or hypoxic-ischemic encephalopathy, and repeated measurements can be useful for tracking clinical improvement [71]. ASL has also been used to identify CBF deficits—some located in anatomically apparently intact regions—in chronic stroke survivors, a finding that may have major implications for the study of stroke-related behavioural deficits [47]. Conversely, regions of hyperperfusion have been seen in patients with a history of ischemic injury, potentially due to loss of autoregulation of cerebral vascular resistance [72]. ASL has also been used for investigations of stroke-like episodes in patients with mitochondrial encephalomyopathy, lactic acidosis, and stroke-like episodes (MELAS), identifying hyperperfusion in the acute phase and hypoperfusion in the chronic phase [73].

Identification of transient ischemic attack (TIA) is essential for optimal patient outcome, as urgent specialised management may reduce the risk of subsequent stroke by 80 % [74], but accuracy of this diagnosis is often difficult. Diffusion weighted imaging (DWI) is usually recommended for evaluation of suspected TIA, but commercially available pCASL software can now increase the diagnostic yield of MRI [75]. Recently, Zaharchuk et al. [76] reported that pCASL was more sensitive to abnormalities in TIA patients than DWI and MR angiography and nearly as sensitive as DSC in identifying TIA lesions. When performed within 24 h, pCASL demonstrated perfusion abnormalities in TIA patients with otherwise normal imaging [77]. Ultimately, pCASL may bolster confidence of diagnosis in post-TIA workup.

For the moment, it has to be said that ASL has a reserve role in the evaluation of (sub)acute ischemic stroke compared to DSC, advantageous only when gadolinium contrast agents are contraindicated. ASL can confirm the presence of regional hypoperfusion—thus ruling out stroke mimics—and rapid identification of the penumbra. Regions with reduced CBF measurements post-stroke have been shown to be largely consistent with regions of DSC hypoperfusion, and one study reported that areas of hyperaemia were more conspicuous on pCASL perfusion-weighted images [78]. This may require further validation, as another group recently found that periprocedural DSC was more sensitive to regional CBF changes after invasive recanalisation [79]. Bokkers et al. [80] found high agreement between pCASL and DSC findings during initial evaluation for stroke. However, ASL may not detect small perfusion deficits as accurately as larger deficits, and utility for acute stroke may be limited to the pCASL sequence: Zaharchuk et al. [81] found that CASL did not always agree with a DWI perfusion-diffusion mismatch paradigm at 1.5-T scanners. Potential benefits of ASL in diagnosing and classifying infarcts include the detection of border zone and cortical lesions [82], and general identification of infarct zone is supported in other studies [83, 84], but the correct definition of penumbra is not fully established [85].

Utility of ASL in evaluating damage and recovery of arterial ischemic stroke has also been demonstrated in paediatric populations [86]. Perinatal and neonatal ischaemic strokes have relatively unclear mechanisms and few treatment options; a recent study employed ASL in attempts to elucidate brain haemodynamics, demonstrating its feasibility in newborns and supporting its inclusion in MRI protocols [87]. Furthermore, Pienaar et al. [88] produced a quantitative measure to associate regional hyperperfusion with decreased DWI diffusion upon neonatal ischemic insult.

#### Chronic ischaemia and cerebrovascular reserve

While ASL in the context of (sub)acute ischaemia has probably only limited value, its role in assessing risks and consequences of chronic cerebrovascular disease is supported by the published literature. In particular, ASL can be used to assess the cerebrovascular reserve and the hypoperfusion syndrome. The cerebrovascular reserve capacity (CRC) describes to what extent cerebral perfusion is able to increase in the context of vasodilatatory challenges, such as with CO_2_ inhalation or acetazolamide injection. In patients with chronic cerebral hypoperfusion, this reserve is limited due to the fact that the vascular bed is already dilated at baseline, and its capacity to dilate due to vasodilatory challenge is exhausted. While at present the CRC is not taken into account to set the indication for carotid artery surgery [89, 90], recent studies indicate that the risk of cerebral infarction in patients with carotid artery stenosis is considerably higher with reduced CRC [91]. CRC assessment can furthermore contribute to risk assessment prior to carotid artery and heart surgery.

ASL is well suited to assess the CRC because measurements can be repeated, are non-invasive, and are quantitative. Exogenous contrast-enhanced techniques, as well as being invasive, suffer from circulating contrast medium for several days, precluding repeated measurements within this time frame.

ASL has been combined with acetazolamide challenge to assess cerebrovascular reactivity [42, 43], allowing for identification of tissue at highest risk for possible stroke [92]. A recent study related ASL measurements to BOLD activity upon acetazolamide challenge, indicating that CBF measurements contain crucial information not identified with BOLD alone [93].

The effects of treatment can be assessed with ASL. For instance, a study comparing the results of carotid angioplasty with stent placement to those of carotid endarterectomy illustrated restoration of collateral blood flow distribution and normalisation of regional CBF [94].

White matter lesions (WML) are one hallmark of small vessel disease yet are also seen in neuroinflammation, gliosis, and other neurodegenerative processes [95, 96]. While it is currently unclear if ASL perfusion quantification can distinguish between WML types, one study reported correlation between perfusion disturbances and the extent of white matter disease, with later-stage WML subjects exhibiting decreased CBF in both white and grey matter [97]. Sensitivity of ASL is reduced in white matter, due to lower CBF and longer ATT than grey matter, but recent methodological advances including higher field strength, pCASL, and sequence optimisation can address that challenge [98, 99].

#### Clinical assessment and interpretation

The diagnosis of ischaemia on ASL is generally based on reduced flow from the proximal routes. Blood flow via collateral routes is difficult to detect as it takes longer, leading to increased delays between the labeling of the spins and their arrival in the imaged voxel. The main problem of ASL in studying acute or chronic cerebral and cardiovascular conditions is related to transit delay and visualisation of collateral flow due to the rapid decay of the magnetic label [5, 100]. Choosing the appropriate PLD for ASL is therefore crucial but as mentioned above is a trade-off against SNR [101].

**Fig. 8:**
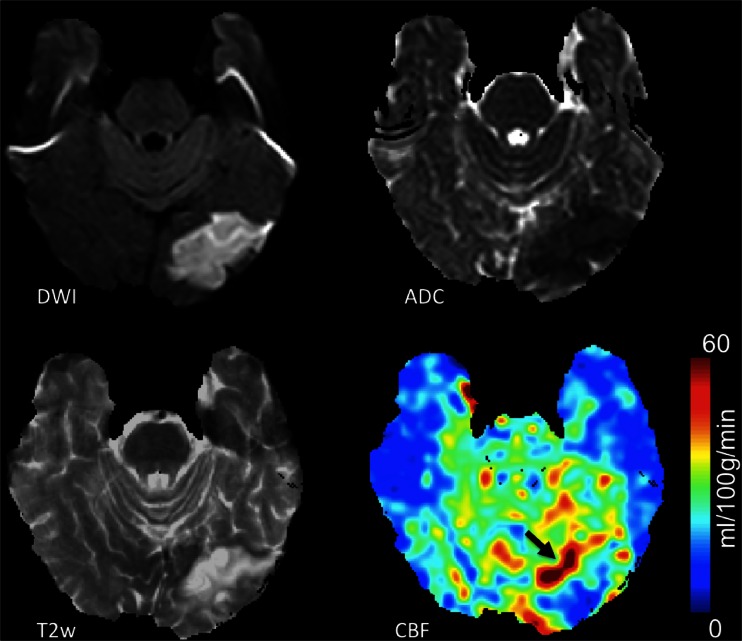
Acute ischaemia in the left occipital lobe, with diffusion restriction on the diffusion-weighted image (*DWI*) and apparent diffusion coefficient (*ADC*) map, and high signal intensity on the T2 weighted (*T2w*) images. The colour-coded cerebral blood flow (*CBF*) map shows hypoperfusion in the ischaemic region, with the *arrow* indicating the residual vascular signal in the arteries feeding the ischaemic tissue (arterial transfer artefact: *ATA*)

When the arterial arrival times are the same as or longer than the PLD, labeled spins can be visualised in the arteries feeding the ischemic tissue, a finding that has been called the arterial transit artefact (ATA) [42]. Recently, Yoo et al. reported high utility of bright vessel appearance due to ATA on pCASL imaging for localising occlusions in acute stroke [102]. This artefact, which in this context is thus in fact an important marker of pathology, is however not visible if a vascular suppression method is applied. Vascular suppression methods have their use when ATA needs to be avoided for optimal CBF quantification [103] but should not be used when ASL is performed for the detection of ischaemia. Low ASL signal, with surrounding cortical areas of high signal intensity due to ATA in the border zones of the middle (MCA) and anterior cerebral artery (ACA) or MCA and posterior cerebral artery (PCA), has been called the ‘border zone sign’. This sign was found to be more sensitive than contrast-enhanced perfusion-weighted imaging for identifying subtle perfusion anomalies [104]. ATA has been correlated with improved outcome after acute stroke [83], possibly reflecting the presence of collateral flow [105] (Fig. 8). ATA was often present surrounding the ischemic core and tended to be associated with lack of progression to infarct and better clinical outcome [86]. One should keep in mind however that ASL tends to overestimate the perfusion deficit and penumbra, especially in patients with small infarct size [106].

**Fig. 9:**
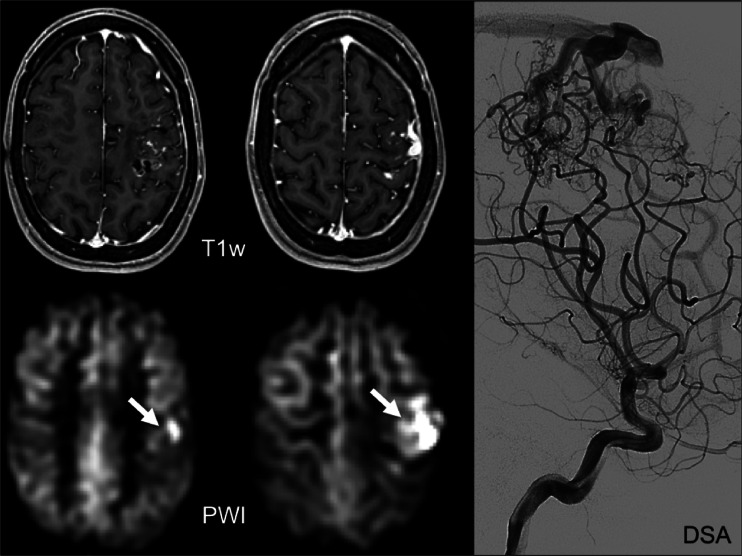
Post-contrast T1 weighted (*T1w*: *top row*) and perfusion-weighted images (*PWI*: *bottom row*) obtained with ASL of an arteriovenous malformation as evidenced by digital subtraction angiography (*right column*: *DSA*). The *arrows* indicate high signal in the draining veins

ASL images are very sensitive to alterations or physiological variants of the brain drainage and circulation (circle of Willis normal variants and developmental venous anomalies), and they can be used to detect AVMs (Fig. 9) or fistulas (AVF), quantify the arteriovenous shunting, and evaluate CBF alteration in the adjacent and distant brain tissue [107]. If a significant asymmetry of brain perfusion is detected on the ASL or CBF maps, it is important to review all the standard MR sequences acquired and to add MR angiography to evaluate or exclude the presence of possible normal variants or of pathologic vascular shunts which can cause the alteration of the brain perfusion.

### Dementia

Diagnosis and evaluation of neurodegenerative disease is a rapidly expanding application of ASL, particularly with recent growth in biomarker exploration for Alzheimer’s disease (AD) and dementia [108]. Not only does the quantification of perfusion offer a role in monitoring disease progression, which is potentially useful for clinical trials of new therapies, it may also be able to provide helpful diagnostic information if characteristic perfusion patterns can be established. Changes in brain metabolism and perfusion often precede observable structural changes such as atrophy in neurodegenerative diseases. This is most commonly assessed with fluor-deoxyglucose (FDG)-PET, which is not always cost-effective [109] and has limited availability, in contrast to ASL, which can be added to the routine structural MRI examination at low incremental cost and only 5 min of additional scan time. CBF measurements derived from ASL correlate with gold standard H2[15O]-PET [110, 111].

AD is the most common cause of dementia [108]. The use of ASL in AD was recently reviewed [112], assessing the technique as an emerging biomarker. Altered metabolic activity measured with FDG-PET has been linked to early pathologic changes in AD patients in multiple studies, and regions of altered perfusion measured with ASL appear to overlap with these findings [113, 114]. A number of studies have yielded interesting perfusion comparisons between patients with various forms of dementia and demographically matched healthy controls. ASL has detected global perfusion decreases in AD patients [115] in addition to regional hypoperfusion. Both AD and mild cognitive impairment (MCI), the intermediate stage between normal cognitive decline and dementia, have been associated with hypoperfusion in the middle occipital areas, medial temporal lobe, and especially the parietal lobe [116]. Similar hypoperfusion has been reported in the posterior cingulate and precuneus in addition to certain frontal and parietal regions [117, 118]. Frontotemporal dementia is also associated with regional hypoperfusion, though the spatial distribution is different from that of AD [119], with bilateral hypoperfusion in the frontal cortex and insula and hyperperfusion in the precuneus and posterior cingulate. ASL is potentially a useful tool for this important differential diagnosis [120, 121].

Regional hypoperfusion as assessed with ASL may be a good predictor of cognitive decline in AD and potentially identify candidates for treatment trials [122]. Regional reductions in CBF have also been correlated with known AD risk factors like the apolipoprotein E epsilon 4 allele, further supporting ASL’s utility in indicating disease progression from MCI to AD [123]. Findings from Dashjamts et al. suggest that ASL imaging is better than morphological imaging (voxel-based grey matter density) in the diagnosis of AD [124], although Bron et al. report no added diagnostic value of ASL over structural MRI atrophy markers in presenile dementia classification [125]. A number of studies utilise ASL as part of a multi-tool approach for elucidating AD-related changes, for instance alongside magnetic resonance spectroscopy [126] or hippocampal volume measurement [127]. Regional reduction of perfusion in AD is not necessarily related to other instances of neuropathological hypoperfusion. Proposed mechanisms for AD and subcortical ischaemic vascular dementia remain controversial [128], with debate as to whether hypoperfusion is a cause or consequence of neurodegeneration [115]. Yoshiura et al. found no correlation between regional ATT prolongation and regional hypoperfusion in AD [129], which suggests a different mechanism than that of cerebrovascular disease. While co-occurring pathologies make it difficult for researchers to agree, vascular abnormalities have recently been suggested to play a critical role in AD pathology given known characteristics of beta-amyloid clearance dysfunction [130].

As biomarker research has greatly expanded over the last two decades, reconciliation of the accumulating findings comes with many considerations [108], particularly the translation of group-level findings to individual patients [131]. Overall, the literature demonstrates both the technical feasibility and advantages of ASL in neurodegenerative disease. Evidence suggests that AD staging models ought to incorporate CBF changes as an early biomarker [132]. The relative low cost and ease of implementation of ASL favour its potential inclusion in methods for long-term surveillance of ageing populations. Wang et al. demonstrated the feasibility and compelling clinical utility of ASL across multiple sites [133].

#### Clinical assessment and interpretation

For the diagnosis of AD, the two regions to scrutinise for hypoperfusion are the precuneus and the posterior cingulate cortex [111, 112, 120, 122, 129, 134–137]. In addition, hypoperfusion in the lateral parietal cortex bilaterally supports the diagnosis [135, 137, 138]. This is essentially not different from the findings with FDG-PET, with which these regions are identified as being hypometabolic. Similar abnormalities, albeit to a lesser extent, can be found in MCI [135, 139, 140]. Note, however, that hyperperfusion can also be observed in MCI, particularly in the hippocampus, amygdala, and striatum [139].

**Fig. 10:**
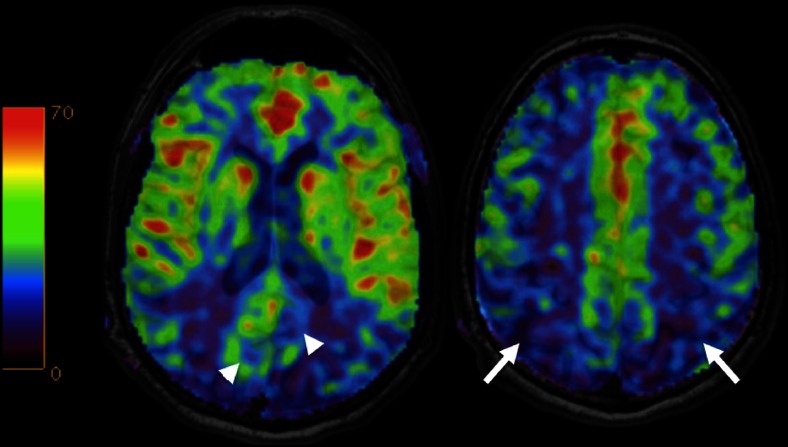
Colour-coded cerebral blood flow maps acquired with ASL overlaid on structural T1w images show hypoperfusion in the precuneus and posterior cingulate cortex (*arrowheads*) and posterior parietal cortex (*arrows*) bilaterally consistent with Alzheimer’s disease

**Fig. 11:**
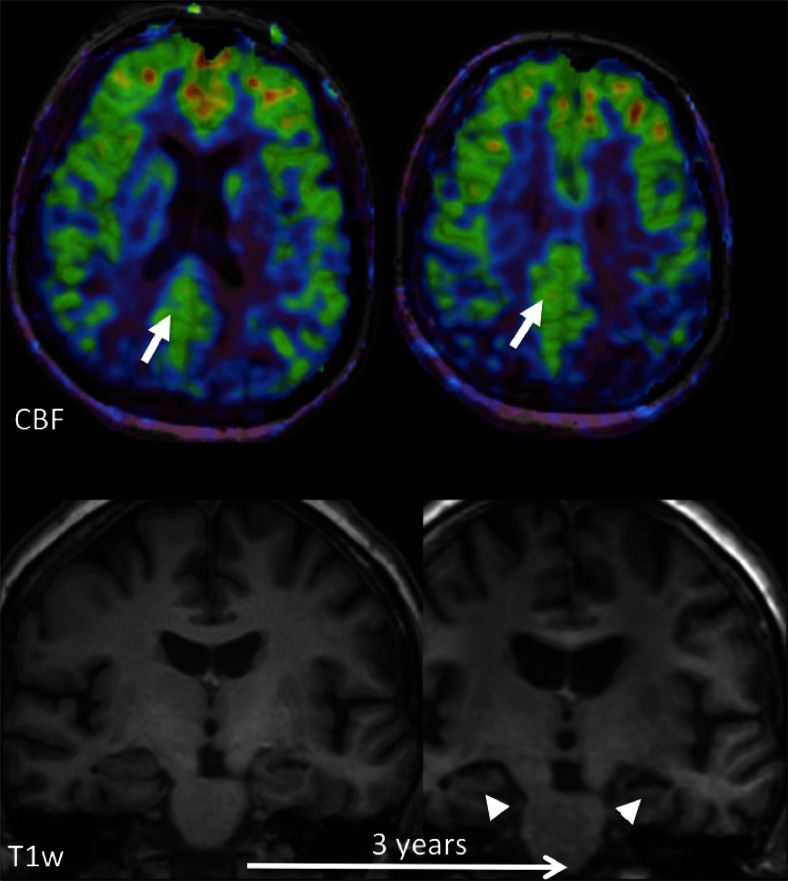
Early perfusion changes in Alzheimer’s disease (*AD*). *Top row*: colour-coded cerebral blood flow (*CBF*) maps acquired with ASL overlaid on structural T1w images at baseline; *bottom row*: coronal reconstructions at the level of the hippocampus at baseline and after 3 years. At baseline, hippocampal volume is normal, but hypoperfusion in the posterior cingulate cortex/precuneus (*arrows*) already indicates AD. Note that the hypoperfusion may easily be missed, as there is no clear perfusion deficit. Perfusion in this area however should be much higher than the rest of the cortex, while here it is similar to the rest of the cortex. This is abnormal. After 3 years, structural changes consistent with AD, i.e., hippocampal and global atrophy, also become visible

An important pitfall in the diagnostic assessment of ASL-perfusion maps for AD and MCI is the fact that the precuneus and posterior cingulate cortex are more highly perfused than the rest of the cerebral cortex [141] (Fig. 10). Mild hypoperfusion thus may not be immediately apparent upon qualitative, visual inspection, as it will not appear as a cortical perfusion deficit. Rather, when perfusion in these regions approaches that of the rest of the cerebral cortex, and is not clearly higher, this should be considered as hypoperfusion and indicative of neurodegenerative pathology (Fig. 11). Clearly, quantitative assessment will be beneficial in detecting such subtle perfusion abnormalities, but unfortunately the lack of reference standards, large inter- and intra-individual variation, and issues with quantification prohibit such an evaluation at the moment.

### Neuro-oncology

Perfusion imaging provides useful information about vascularisation and vascular proliferation, which is directly applicable to the assessment of brain tumours. Tumour growth requires a substantial supply of blood. Angiogenesis occurs once neoplastic tissue reaches a critical mass, and the particular function and architecture of new blood vessels are linked to tumour type [142]. While relative cerebral blood volume (CBV) measured by DSC perfusion MR imaging is the most common measurement, CBF measured by ASL perfusion imaging has comparable utility in tumour diagnosis, grading, and follow-up of tumours and treatment.

While it may not be appropriate to compare perfusion values determined with different techniques [142], multiple studies seem to indicate that DSC and ASL findings correlate well. Although DSC perfusion is more widely used to evaluate brain tumours, Lehmann et al. [143] found that pCASL detected gliomas, metastases, and meningiomas on a 3 T scanner as accurately as DSC, and Järnum et al. [144] reported a similar correlation for a variety of tumour classifications. More recently, Hirai et al. [145] found that ASL measurements nearly matched DSC quantifications of regional CBF in gliomas, and van Westen et al. [146] reported ASL and DSC blood volume correlation for various intracranial tumours.

Pretreatment classification of tumour types can be hugely important for timely and effective intervention. A recent study found that ASL performed as well as DWI and FDG-PET in distinguishing primary central nervous system lymphomas (PCNSLs) from glioblastoma, identifying the denser and less vascular PCNSLs due to decreased perfusion [147]. This example demonstrates the strategy for using ASL to differentiate tumour types: correlating known histological characteristics with perfusion measurements to yield information otherwise collected by more invasive means. A number of other studies have illustrated this approach. Noguchi et al. [148] found statistically significant differences in ASL-quantified tumour perfusion when comparing haemangioblastomas to gliomas, meningiomas, and schwannomas due to the tumour types’ different vascular densities. Three-tesla PASL has been used to differentiate haemangioblastomas and metastatic tumours, which are the major differential diagnoses of tumours in the posterior fossa in adults [149]. Recently, PASL showed high accuracy in distinguishing pilomyxoid astrocytomas (a relatively new WHO Classification addition) from pilocytic astrocytomas [150]. This type of differentiation is essential for making therapeutic decisions.

Tumour grading is another important informant of treatment and is partly determined by the extent of vascular proliferation. High- and low-grade gliomas can be distinguished on the basis of perfusion quantification with CASL [151, 152] and PASL [153–156]. Relative CBF measured with ASL has been shown to correlate with relative CBV measured with DSC [157]. Generally, high-grade tumours exhibit CBF above the individual mean, while low-grade tumours exhibit CBF below the mean [153].

Follow-up of tumours with perfusion imaging can greatly inform management. Adult low-grade tumours undergo malignant transformation, which is characterised by a switch from avascular to vascular tumour, known as the angiogenic switch. This is yet to be measured with ASL. Perfusion in high-grade tumours can be measured with ASL before and after treatment to assess response and progression. This is useful in the context of anti-angiogenic treatment: in a case report of a patient treated for recurrent glioblastoma, ASL illustrated tumour progression before conventional MRI did [158]. ASL was furthermore reported to be more effective than DSC at distinguishing radiotherapy-induced necrosis from high-grade glioma recurrence (sensitivity >90 %) [159].

#### Clinical assessment and interpretation

ASL is not yet commonly used for brain tumour assessment and diagnosis, presumably due to the fact that contrast is generally administered for this indication. ASL as a non-invasive technique is thus often dismissed as irrelevant, but this may not always be justified. Particularly in the paediatric population, patients after chemotherapy with difficult intravenous access, and cases of renal insufficiency, a non-invasive technique without exogenous contrast administration may be preferred. A clear advantage of ASL is that CBF quantification is not affected by T1 and T2 leakage effects with blood–brain barrier disruption, which is a major issue with contrast-enhanced perfusion techniques. Furthermore, depending on the readout sequence used, susceptibility artefacts are not necessarily an issue with ASL. The assessment of CBF in brain tumours is not different from contrast-enhanced perfusion techniques, although quantitative thresholds are not yet widely established.

## Considerations and limitations

### Quality control

**Fig. 12:**
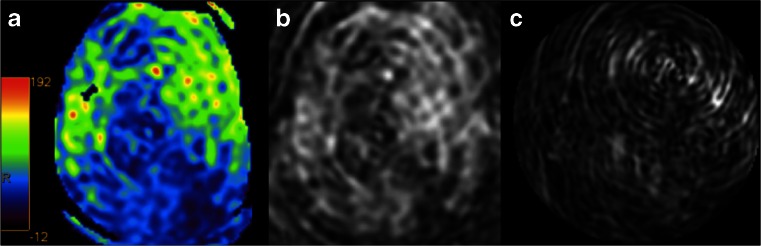
Colour-coded CBF map (**a**) with severe motion artefacts. The artefacts can easily be appreciated on the source images (**b**, **c**) as linear and spiral patterns

**Fig. 13:**
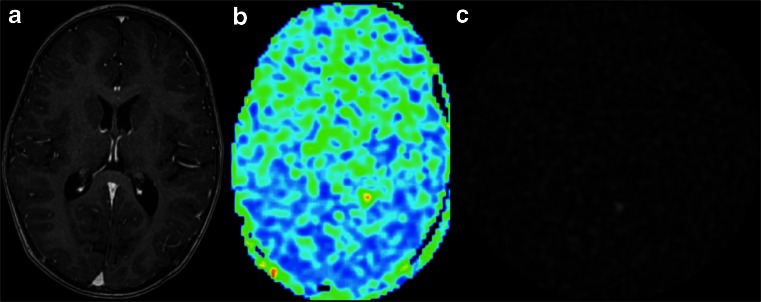
ASL acquisition—inadvertently—after contrast administration (**a**). While the post-processing software will provide a ‘CBF’ map (**b**), the source images (**c**) clearly show random noise without any signal

As with any imaging exam, quality control is essential prior to clinical assessment. Common artefacts include motion, signal dropout, distortion, bright spots, and labeling failure [160]. Motion artefacts may appear as rings or curved lines and may result in artefactually high or low CBF values (Fig. 12). Signal dropout and distortion result from susceptibility effects with EPI-based readout sequences. These typically occur at air–tissue interfaces, such as near the frontal sinuses or mastoid bone. Metallic surgical material and haemorrhage are additional sources of such artefacts. Bright spots are random—clusters of—voxels of very high perfusion, due to residual vascular signal. As described above, note that this artefact is in fact of diagnostic value as a feature of slow flow in acute ischaemic stroke. Failure to label the inflowing blood, e.g. due to local susceptibility artefacts, results in apparent lack of perfusion in the entire affected vascular territory (Fig. 7). Finally, ASL should never be acquired after the administration of gadolinium-based contrast, as the resulting T1-shortening is detrimental for the label (Fig. 13). Note that this effect is noticeable for days after contrast administration.

### Confounds

Brain perfusion is highly dynamic and will be influenced by many factors [161]. Some factors are likely to generate long-lasting global and local perfusion adaptations. These include genetics, cognitive capacity [162], personality traits [163], physical exercise [164], and age [165]. Other factors are much more variable and define a state of mind at the time of examination. These include for instance mood [166], blood gases [167], nutrition [168], stress [169], and medication use. Some of the induced changes are global and related to vascular tonus, while other local variations are the results of psychotropic effects on the brain. For the interpretation of perfusion maps and derived values, such variance should ideally be taken into account. Some changes induced by physiology, state of mind, and non-metabolism-related factors may confound interpretation related to disease states, because such changes are in the same areas and of a comparable magnitude. Especially early abnormalities might be obscured by the noise introduced by confounds and modifiers of perfusion. It is therefore suggested that studies of perfusion of the brain always inquire about possible confounds and modifiers using a questionnaire. This information can then be used to understand the validity of perfusion alterations. Where possible, corrections can be applied to the quantitative results by measuring the modifier, e.g. blood gases and haematocrit. The use of a standard operating procedure while performing the perfusion measurement is also advisable to reduce the influence of, e.g. time of day, wakefulness/consciousness, satiety, acute substance use, and the use of certain prescription drugs. As an example, drinking a cup of coffee or smoking a cigarette just before the perfusion measurement has substantial influence on both global and local quantification [170, 171].

### Paediatric population

As previously mentioned, ASL is particularly effective for imaging paediatric populations. Non-invasive imaging avoids ethical controversy over the injection of contrast agents, and children have a higher baseline CBF signal, which results in a better signal-to-noise ratio [4]. Repeat measurements, the accuracy of which has been experimentally demonstrated, are useful in neurodevelopmental studies of children [172] and sustained monitoring in high-risk neonates, particularly in attempts to identify factors linked to brain injury in development [173]. Though applications of ASL in epilepsy, neoplasms, and other neurological disorders have not been fully evaluated for paediatric groups, the same principles apply. Studies have demonstrated the utility of ASL in evaluating perfusion changes associated with sickle cell disease, the most common cause of stroke in children [174]. However, care must be taken to not overestimate CBF abnormalities. Since most ASL studies are done in adult populations, sequences must sometimes be optimised for a younger population with different average physiological parameters [175].

### Quantification

ASL’s ability to quantify CBF is its major advantage over contrast-enhanced MR perfusion techniques. Quantitative assessment of CBF potentially allows for the use of threshold or reference values, thus aiding in the—more objective—differentiation between healthy and diseased conditions. Such an assessment is less user- and experience-dependent and more sensitive to subtle alterations than visual assessment.

CBF is commonly quantified according to the following equation for (p)CASL [21]:

$$ \mathrm{C}\mathrm{B}\mathrm{F}=\frac{6,000\cdot \lambda \cdot \left({M}_{\mathrm{c}}-{M}_{\mathrm{l}}\right)\cdot \frac{\mathrm{PLD}}{e^{T_{1\mathrm{Blood}}}}}{2\cdot \alpha \cdot {T}_{1\mathrm{Blood}}\cdot {M}_{\mathrm{PD}}\left(1-{e}^{\left(\frac{\tau }{T_{1\mathrm{Blood}}}\right)}\right)} $$

where *λ* is the brain/blood partition coefficient in millilitres per gram, *α* is the labeling efficiency, *M*_c_ is the control and *M*_l_ is the labeled signal intensity, and *M*_PD_ is the signal intensity of a proton density-weighted image. This is used to normalise the overall signal intensity which potentially varies due to several confounders, such as hardware and patient variability globally affecting the signal. Recommendations on how to obtain *M*_PD_ can be found in Alsop et al. [16]. Six thousand is a conversion factor of the units from ml/g/s to ml/100 g/min. While many of the parameters can be estimated, it is common to use nominal values indicating that quantification is still partially based on assumptions. Nominal values are as follows: *λ* = 0.9 ml/g [176], *T*_1Blood_ = 1,650 ms at 3.0 T [177] and 1,350 ms at 1.5 T [178], *α* = 0.85 for pCASL [179], and 0.98 for PASL [180].

**Fig. 14:**
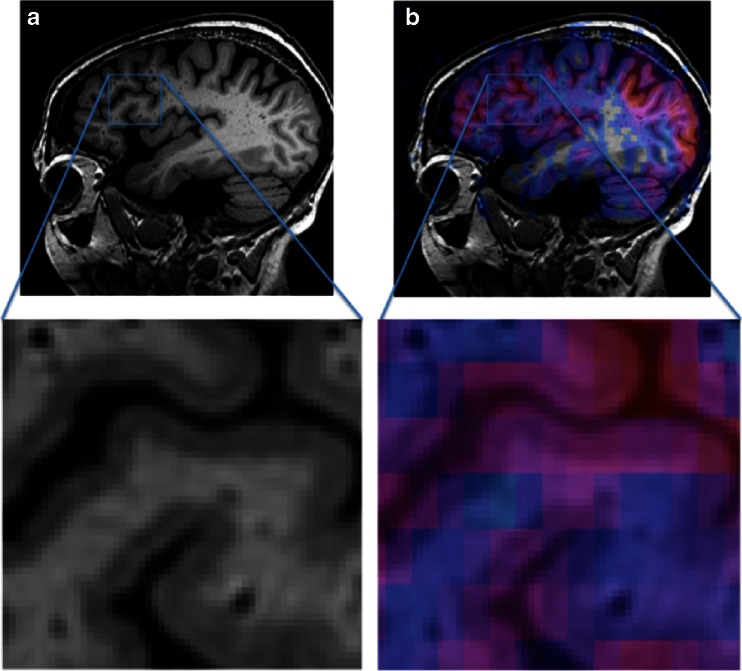
Zoomed in sagittal views of high-resolution T1-weighted images without (**a**) and with (**b**) CBF map overlay. Coloured ASL voxels include grey matter, white matter, and cerebrospinal fluid simultaneously, illustrating the partial volume effect. Partial volume effects can be dealt with by using a post-processing technique called partial volume correction [181]

An important aspect affecting the quantitative assessment of CBF is the partial volume effect, related to the fact that the voxel size of ASL is several times that of 3D T1-weighted acquisitions (Fig. 14). Hence, most voxels contain a combination of tissues and/or cerebrospinal fluid. Different tissues have substantially different perfusion characteristics. Perfusion in the white matter is about half that of grey matter, while at the same time T1 is shorter and arterial arrival time is longer. The partial volume effect becomes even more relevant in the presence of atrophy, when voxels contain relatively less grey matter and subsequently lower average CBF. Such unwanted effects are dealt with by using a post-processing technique called partial volume correction [181].

## Emerging applications and techniques

For the neurological diseases discussed above, current state-of-the-art pCASL imaging is ready for clinical implementation. There are several more applications in which perfusion imaging with ASL has potential value, but these are still more or less in the phase of assessment. These include neurodegenerative diseases such as Parkinson’s [182, 183] and Huntington’s disease [184, 185], multiple sclerosis [186–188], epilepsy, and psychiatric disease. Below we highlight the latter two.

Regarding the technical developments related to ASL, we already touched upon the value and issues of imaging at high field strength. Ultrahigh field strength (7 T or higher) makes long PLDs (as long as 6 s) feasible, thereby drastically improving ASL’s ability to visualise regions with delayed flow and collateral blood flow [35, 189]. This also opens up the possibility of more accurate CBF measurement in areas of relatively low perfusion such as the white matter and arterial border zones. Quantification of CBF is further improved at high field strength by the reduction of partial volume effects with improved spatial resolution [190]. SNR increases approximately fourfold from 3 to 7 T [191]. High SNR in turn allows imaging at increased spatial resolution (1–2-mm^2^ in-plane resolution) [191]. Imaging at multiple PLDs, effectively mapping and quantifying arrival times into the tissue [51, 192, 193], again is an area in which ultrahigh field strengths will be beneficial, because this technique is currently limited by low SNR, persistent inaccuracies in regions with severely delayed flow, and challenges with non-linear fitting of CBF to the kinetic models [21].

A further technical development that we describe here is territorial ASL, which has great potential for the functional assessment of cerebrovascular disease.

While research studies in both these emerging applications and techniques are interesting and show promising results, proper clinical evaluation is still needed for assessing their true value and validity, and further research is warranted.

### Epilepsy

In the evaluation of epilepsy, SPECT and PET are used to measure ictal and interictal blood flow for localisation of epileptogenic activity, illustrating increased perfusion in the critical period and decreased perfusion after seizures [194]. However, nuclear medicine techniques can be expensive or contraindicated. ASL can potentially assist clinical diagnosis by quickly identifying potential perfusion asymmetries or ruling out causes such as stroke. Non-invasive measurements could be repeated to assess changes over time, particularly in response to treatment. Repeated seizures can lead to a chronic epileptic condition, demonstrated experimentally [195] and clinically [196], and it is thought that neuroinflammation and seizure-induced vascular alterations contribute to epileptogenesis [197], warranting investigation of such changes with perfusion imaging.

A few studies have employed interictal [198–200] and immediate post-ictal [201, 202] PASL measurements in cases of partial epilepsy. Correlation between ASL, PET, and electrophysiological data has been shown in patients with interictal hypoperfusion in surgically intractable epilepsy [203] and tuberous sclerosis [204]. PASL also compares favourably to PET and electrical source imaging in identifying the epileptogenic zone [205], indicating diagnostic value and potential utility in guiding intracranial electrode placement. Recent work demonstrated comparable measurements between PASL and DSC for detecting seizure-associated perfusion asymmetries [206].

Ictal ASL measurements have only been reported in case studies, as seizures must coincidentally occur during scans. One patient’s complex partial seizure during PASL acquisition showed ictal hyperperfusion in the typical seizure focus, a finding that was not present interictally [207]. This matches the underlying pathophysiological mechanism of a seizure; excess activation requires increased blood flow to deliver glucose and oxygen. Oishi et al. [208] captured PASL images of a patient during and after partial status epilepticus. The case study followed the same trends, with marked hyperperfusion in the epileptogenic zone during the seizures and hypoperfusion compared to the non-affected hemisphere once the seizures stopped. Finally, Kanzawa et al. report that the combined use of ASL and DWI was useful in differentiating non-convulsive partial status epilepticus from stroke when ictal discharges were not captured with EEG, leading to appropriate treatment and resolution of symptoms [209].

Ictal perfusion imaging provides better information than interictal imaging alone [210]. Since ictal SPECT only depends on injection time whereas ictal ASL requires simultaneous scanning, which is not typically compatible with electroencephalography, regular utility of ASL is currently limited to interictal foci lateralisation. Perfusion quantification can also yield valuable insight into seizure-causing lesions such as cortical dysplasia [211]. Identification of such lesions is vital for pre-surgical planning and operative outcome.

### Psychiatric disease

The use of ASL in exploring the neurobiological and anatomical correlates of clinical psychiatric conditions is steadily increasing. It is thought that cerebral microvasculature abnormalities may play a role in some psychiatric illnesses, the mechanisms of which are poorly understood [212], but changes in regional CBF are generally attributed to changes in neuronal activity and energy demand secondary to psychiatric disease or its therapy.

Baseline differences in CBF between patients and healthy controls have been identified in several ASL studies. Chronic and treatment-resistant depression showed associated perfusion abnormalities in the pathologically relevant subgenual anterior cingulate cortex compared to healthy controls [213]. Adolescent depression appears to affect regional CBF measurements in executive, affective, and motor networks [214]. Late-life depression has been associated with elevation of white matter CBF [215]. Schizophrenia has been correlated with hypoperfusion of the prefrontal cortex [216–218], in addition to complex patterns of altered perfusion in other brain areas. Borderline personality disorder was associated with decreased CBF in the medial orbitofrontal cortex and increased CBF in the lateral orbitofrontal cortex compared to healthy controls [219].

ASL may also be used to assess treatments. Regional CBF measurements have been shown to predict response of patients with depression [220] and schizophrenia [221] to repetitive transcranial magnetic stimulation, indicating potential for individualised therapy. Studies isolating the effects of pharmaceuticals on brain perfusion in healthy subjects allow for greater understanding of their application in psychiatric conditions, where changes in regional perfusion can complicate such assessment [222–224].

### Territorial ASL

**Fig. 15:**
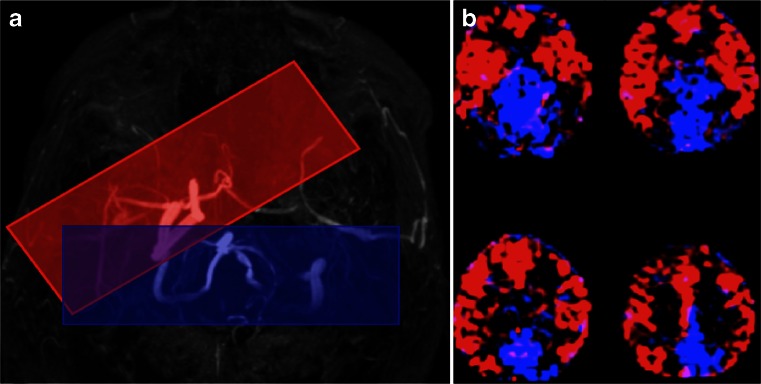
Positioning of the selective labeling planes for territorial ASL based on an MR angiogram of the circle of Willis for the right internal carotid artery (*ICA*, *red box*) and the basilar artery (*blue box*). The labeling plane for the left ICA is not shown, as this vessel was occluded and no signal was obtained. **b** Colour territorial perfusion maps showing the distribution of brain tissue perfused by the intracranial arteries labeled in (**a**). The anterior and middle cerebral artery territories in both hemispheres are supplied by the right ICA in this patient with left ICA occlusion (in *red*). The medial occipital and parietal lobes are supplied by the basilar artery (in *blue*)

Territorial ASL refers to the ASL method that provides a perfusion measurement from a specific vascular territory (Fig. 15). The original methods, based on PASL sequences, allowed for a separation mostly of each of the two perfusion territories from the internal carotid arteries (ICAs), and the posterior territory, without distinction between ACA and MCA territories or between left and right vertebral arteries [225]. More recent methods, based on pCASL, allow for a much finer assessment of the perfusion territories from smaller arteries, with the caveat that separate measurements from the ACA and MCA will require imaging of perfusion in tissues distal to the circle of Willis and will therefore never be able to produce whole brain perfusion maps [226]. For an extensive review on all territorial ASL methods, the reader is directed to Hartkamp et al. [227]. Territorial ASL has been used in many studies of sub-acute stroke and has shown to provide additional information on the underlying pathology of lateralised lesions. In particular, it has been shown that in about 10 % of patients with a cortical lesion, assessment of the anatomical location alone results in a misclassification of the perfusion territory in which the infarct is located. The availability of a vascular territorial perfusion map allows to correct this [82]. An additional potential application of territorial ASL is in the context of steno-occlusive disease, when both information on the affected vessel’s perfusion territory and an assessment of cerebrovascular reserve capacity can be obtained.

## Conclusion

ASL is now commercially available on MRI systems from all major vendors and is increasingly making its way into clinical practice. Its non-invasive and quantitative nature makes the technique especially attractive for vulnerable patient populations, such as the elderly, children, oncological patients with difficult venous access, and patients with renal insufficiency. There is currently sufficient evidence to support its clinical application in dementia, neuro-oncology and cerebrovascular disease, with clear advantages in terms of improved and earlier diagnosis. With the current tremendous research efforts in other areas of neurological and psychiatric disease, it can be expected that additional clinical indications will arise in the near future.

## References

[1] Wintermark M, Sesay M, Barbier E (2005). Comparative overview of brain perfusion imaging techniques. Stroke.

[2] Williams DS, Detre JA, Leigh JS, Koretsky AP (1992). Magnetic resonance imaging of perfusion using spin inversion of arterial water. Proc Natl Acad Sci U S A.

[3] Sadowski EA, Bennett LK, Chan MR (2007). Nephrogenic systemic fibrosis: risk factors and incidence estimation. Radiology.

[4] Wang J, Licht DJ, Jahng G-H (2003). Pediatric perfusion imaging using pulsed arterial spin labeling. J Magn Reson Imaging.

[5] Wolf RL, Detre JA (2007). Clinical neuroimaging using arterial spin-labeled perfusion magnetic resonance imaging. Neurotherapeutics.

[6] Gevers S, Majoie CBLM, van den Tweel XW (2009). Acquisition time and reproducibility of continuous arterial spin-labeling perfusion imaging at 3T. AJNR Am J Neuroradiol.

[7] Jiang L, Kim M, Chodkowski B (2010). Reliability and reproducibility of perfusion MRI in cognitively normal subjects. Magn Reson Imaging.

[8] Wang Y, Saykin AJ, Pfeuffer J (2011). Regional reproducibility of pulsed arterial spin labeling perfusion imaging at 3T. Neuroimage.

[9] Mutsaerts HJMM, Steketee RME, Heijtel DFR (2014). Inter-vendor reproducibility of pseudo-continuous arterial spin labeling at 3 tesla. PLoS One.

[10] Golay X, Hendrikse J, Lim TCC (2004). Perfusion imaging using arterial spin labeling. Top Magn Reson Imaging.

[11] Petersen ET, Zimine I, Ho Y-CL, Golay X (2006). Non-invasive measurement of perfusion: a critical review of arterial spin labelling techniques. Br J Radiol.

[12] Liu TT, Wong EC, Buxton RB, Squire E-IR (2009). Perfusion MRI. Encyclopedia of neuroscience.

[13] Golay X, Guenther M (2012). Arterial spin labelling: final steps to make it a clinical reality. MAGMA.

[14] Detre JA, Rao H, Wang DJJ (2012). Applications of arterial spin labeled MRI in the brain. J Magn Reson Imaging.

[15] Essig M, Shiroishi MS, Nguyen TB (2013). Perfusion MRI: the five most frequently asked technical questions. AJR Am J Roentgenol.

[16] Alsop DC, Detre JA, Golay X (2014). Recommended implementation of arterial spin-labeled perfusion MRI for clinical applications: a consensus of the ISMRM perfusion study group and the European consortium for ASL in dementia. Magn Reson Med.

[17] Maccotta L, Detre JA, Alsop DC (1997). The efficiency of adiabatic inversion for perfusion imaging by arterial spin labeling. NMR Biomed.

[18] Utting JF, Thomas DL, Gadian DG, Ordidge RJ (2003). Velocity-driven adiabatic fast passage for arterial spin labeling: results from a computer model. Magn Reson Med.

[19] Gach HM, Dai W (2004). Simple model of double adiabatic inversion (DAI) efficiency. Magn Reson Med.

[20] Trampel R, Jochimsen TH, Mildner T (2004). Efficiency of flow-driven adiabatic spin inversion under realistic experimental conditions: a computer simulation. Magn Reson Med.

[21] Buxton RB, Frank LR, Wong EC (1998). A general kinetic model for quantitative perfusion imaging with arterial spin labeling. Magn Reson Med.

[22] Wong EC (2014). An introduction to ASL labeling techniques. J Magn Reson Imaging.

[23] Henkelman RM, Huang X, Xiang QS (1993). Quantitative interpretation of magnetization transfer. Magn Reson Med.

[24] Detre JA, Leigh JS, Williams DS, Koretsky AP (1992). Perfusion imaging. Magn Reson Med.

[25] Pizzini F, Smits M, Wesolowski R, Saremi F (2015). Arterial spin labeling. Advances in noninvasive perfusion imaging: a multimodality diagnostic approach to tissue perfusion analysis.

[26] Kim SG (1995). Quantification of relative cerebral blood flow change by flow-sensitive alternating inversion recovery (FAIR) technique: application to functional mapping. Magn Reson Med.

[27] Wong EC, Buxton RB, Frank LR (1997). Implementation of quantitative perfusion imaging techniques for functional brain mapping using pulsed arterial spin labeling. NMR Biomed.

[28] Edelman RR, Chen Q (1998). EPISTAR MRI: multislice mapping of cerebral blood flow. Magn Reson Med.

[29] Garcia DM, Duhamel G, Alsop DC (2005). Efficiency of inversion pulses for background suppressed arterial spin labeling. Magn Reson Med.

[30] Oshio K, Feinberg DA (1991). GRASE (gradient- and spin-echo) imaging: a novel fast MRI technique. Magn Reson Med.

[31] Mulkern RV, Wong ST, Winalski C, Jolesz FA (1990). Contrast manipulation and artifact assessment of 2D and 3D RARE sequences. Magn Reson Imaging.

[32] Vidorreta M, Wang Z, Rodríguez I (2013). Comparison of 2D and 3D single-shot ASL perfusion fMRI sequences. Neuroimage.

[33] Pruessmann KP, Weiger M, Scheidegger MB, Boesiger P (1999). SENSE: sensitivity encoding for fast MRI. Magn Reson Med.

[34] Lu H, Nagae-Poetscher LM, Golay X (2005). Routine clinical brain MRI sequences for use at 3.0 tesla. J Magn Reson Imaging.

[35] Wang J, Alsop DC, Li L (2002). Comparison of quantitative perfusion imaging using arterial spin labeling at 1.5 and 4.0 tesla. Magn Reson Med.

[36] Franke C, van Dorsten FA, Olah L (2000). Arterial spin tagging perfusion imaging of rat brain: dependency on magnetic field strength. Magn Reson Imaging.

[37] St Lawrence KS, Wang J (2005). Effects of the apparent transverse relaxation time on cerebral blood flow measurements obtained by arterial spin labeling. Magn Reson Med.

[38] Biagi L, Abbruzzese A, Bianchi MC (2007). Age dependence of cerebral perfusion assessed by magnetic resonance continuous arterial spin labeling. J Magn Reson Imaging.

[39] Deibler AR, Pollock JM, Kraft RA (2008). Arterial spin-labeling in routine clinical practice, part 2: hypoperfusion patterns. AJNR Am J Neuroradiol.

[40] Deibler AR, Pollock JM, Kraft RA (2008). Arterial spin-labeling in routine clinical practice, part 3: hyperperfusion patterns. AJNR Am J Neuroradiol.

[41] Detre JA, Alsop DC, Vives LR (1998). Noninvasive MRI evaluation of cerebral blood flow in cerebrovascular disease. Neurology.

[42] Detre JA, Samuels OB, Alsop DC (1999). Noninvasive magnetic resonance imaging evaluation of cerebral blood flow with acetazolamide challenge in patients with cerebrovascular stenosis. J Magn Reson Imaging.

[43] Kamano H, Yoshiura T, Hiwatashi A (2013). Arterial spin labeling in patients with chronic cerebral artery steno-occlusive disease: correlation with 15O-PET. Acta Radiol.

[44] Gevers S, Bokkers RP, Hendrikse J (2012). Robustness and reproducibility of flow territories defined by planning-free vessel-encoded pseudocontinuous arterial spin-labeling. AJNR Am J Neuroradiol.

[45] MacIntosh BJ, Lindsay AC, Kylintireas I (2010). Multiple inflow pulsed arterial spin-labeling reveals delays in the arterial arrival time in minor stroke and transient ischemic attack. AJNR Am J Neuroradiol.

[46] Parkes LM, Rashid W, Chard DT, Tofts PS (2004). Normal cerebral perfusion measurements using arterial spin labeling: reproducibility, stability, and age and gender effects. Magn Reson Med.

[47] Brumm KP, Perthen JE, Liu TT (2010). An arterial spin labeling investigation of cerebral blood flow deficits in chronic stroke survivors. Neuroimage.

[48] Bokkers RPH, Bremmer JP, van Berckel BNM (2010). Arterial spin labeling perfusion MRI at multiple delay times: a correlative study with H(2)(15)O positron emission tomography in patients with symptomatic carotid artery occlusion. J Cereb Blood Flow Metab.

[49] Macintosh BJ, Marquardt L, Schulz UG (2012). Hemodynamic alterations in vertebrobasilar large artery disease assessed by arterial spin-labeling MR imaging. AJNR Am J Neuroradiol.

[50] Martin SZ, Madai VI, von Samson-Himmelstjerna FC (2015). 3D GRASE pulsed arterial spin labeling at multiple inflow times in patients with long arterial transit times: comparison with dynamic susceptibility-weighted contrast-enhanced MRI at 3 tesla. J Cereb Blood Flow Metab.

[51] Hendrikse J, van Osch MJP, Rutgers DR (2004). Internal carotid artery occlusion assessed at pulsed arterial spin-labeling perfusion MR imaging at multiple delay times. Radiology.

[52] Kimura H, Kado H, Koshimoto Y (2005). Multislice continuous arterial spin-labeled perfusion MRI in patients with chronic occlusive cerebrovascular disease: a correlative study with CO2 PET validation. J Magn Reson Imaging.

[53] Bokkers RPH, van der Worp HB, Mali WPTM, Hendrikse J (2009). Noninvasive MR imaging of cerebral perfusion in patients with a carotid artery stenosis. Neurology.

[54] Uchihashi Y, Hosoda K, Zimine I (2011). Clinical application of arterial spin-labeling MR imaging in patients with carotid stenosis: quantitative comparative study with single-photon emission CT. AJNR Am J Neuroradiol.

[55] Yun TJ, Sohn C-H, Han MH (2013). Effect of carotid artery stenting on cerebral blood flow: evaluation of hemodynamic changes using arterial spin labeling. Neuroradiology.

[56] Zaharchuk G, Do HM, Marks MP (2011). Arterial spin-labeling MRI can identify the presence and intensity of collateral perfusion in patients with moyamoya disease. Stroke.

[57] Donahue MJ, Ayad M, Moore R (2013). Relationships between hypercarbic reactivity, cerebral blood flow, and arterial circulation times in patients with moyamoya disease. J Magn Reson Imaging.

[58] Noguchi T, Kawashima M, Irie H (2011). Arterial spin-labeling MR imaging in moyamoya disease compared with SPECT imaging. Eur J Radiol.

[59] Goetti R, Warnock G, Kuhn FP (2014). Quantitative cerebral perfusion imaging in children and young adults with moyamoya disease: comparison of arterial spin-labeling-MRI and H2[15O]-PET. AJNR Am J Neuroradiol.

[60] Margariti P, Sanchez-Montanez A, Delgado I (2013). At-risk brain tissue identified with arterial spin labeling in neurotuberculosis. Pediatr Radiol.

[61] Ishitobi M, Yoneda M, Ikawa M (2013). Hashimoto’s encephalopathy with hippocampus involvement detected on continuous arterial spin labeling. Psychiatry Clin Neurosci.

[62] Fiehler J, Illies T, Piening M (2009). Territorial and microvascular perfusion impairment in brain arteriovenous malformations. AJNR Am J Neuroradiol.

[63] Suazo L, Foerster B, Fermin R (2012). Measurement of blood flow in arteriovenous malformations before and after embolization using arterial spin labeling. Interv Neuroradiol.

[64] Amponsah K, Ellis TL, Chan MD (2012). Retrospective analysis of imaging techniques for treatment planning and monitoring of obliteration for gamma knife treatment of cerebral arteriovenous malformation. Neurosurgery.

[65] Shimizu K, Kosaka N, Yamamoto T, et al. (2014) Arterial spin labeling perfusion-weighted MRI for long-term follow-up of a cerebral arteriovenous malformation after stereotactic radiosurgery. Acta Radiol Short Rep 3(1) 10.1177/2047981613510160 PMC4001427 24778796

[66] Yu SL, Wang R, Wang S (2014). Accuracy of vessel-encoded pseudocontinuous arterial spin-labeling in identification of feeding arteries in patients with intracranial arteriovenous malformations. AJNR Am J Neuroradiol.

[67] Aoyama K, Fushimi Y, Okada T (2012). Detection of symptomatic vasospasm after subarachnoid haemorrhage: initial findings from single time-point and serial measurements with arterial spin labelling. Eur Radiol.

[68] Saida T, Masumoto T, Nakai Y (2012). Moyamoya disease: evaluation of postoperative revascularization using multiphase selective arterial spin labeling MRI. J Comput Assist Tomogr.

[69] Zhao WG, Luo Q, Jia JB, Yu JL (2013). Cerebral hyperperfusion syndrome after revascularization surgery in patients with moyamoya disease. Br J Neurosurg.

[70] Zheng G, Zhang LJ, Wang Z (2012). Changes in cerebral blood flow after transjugular intrahepatic portosystemic shunt can help predict the development of hepatic encephalopathy: an arterial spin labeling MR study. Eur J Radiol.

[71] Liu AA, Voss HU, Dyke JP (2011). Arterial spin labeling and altered cerebral blood flow patterns in the minimally conscious state. Neurology.

[72] Pollock JM, Whitlow CT, Deibler AR (2008). Anoxic injury-associated cerebral hyperperfusion identified with arterial spin-labeled MR imaging. AJNR Am J Neuroradiol.

[73] Wang Z, Xiao J, Xie S (2012). MR evaluation of cerebral oxygen metabolism and blood flow in stroke-like episodes of MELAS. J Neurol Sci.

[74] Rothwell PM, Giles MF, Chandratheva A (2007). Effect of urgent treatment of transient ischaemic attack and minor stroke on early recurrent stroke (EXPRESS study): a prospective population-based sequential comparison. Lancet.

[75] Kleinman JT, Zaharchuk G, Mlynash M (2012). Automated perfusion imaging for the evaluation of transient ischemic attack. Stroke.

[76] Zaharchuk G, Olivot J-M, Fischbein NJ (2012). Arterial spin labeling imaging findings in transient ischemic attack patients: comparison with diffusion- and bolus perfusion-weighted imaging. Cerebrovasc Dis.

[77] Qiao XJ, Salamon N, Wang DJJ (2013). Perfusion deficits detected by arterial spin-labeling in patients with TIA with negative diffusion and vascular imaging. AJNR Am J Neuroradiol.

[78] Wang DJJ, Alger JR, Qiao JX (2012). The value of arterial spin-labeled perfusion imaging in acute ischemic stroke: comparison with dynamic susceptibility contrast-enhanced MRI. Stroke.

[79] Nael K, Meshksar A, Liebeskind DS (2013). Periprocedural arterial spin labeling and dynamic susceptibility contrast perfusion in detection of cerebral blood flow in patients with acute ischemic syndrome. Stroke.

[80] Bokkers RPH, Hernandez DA, Merino JG (2012). Whole-brain arterial spin labeling perfusion MRI in patients with acute stroke. Stroke.

[81] Zaharchuk G, El Mogy IS, Fischbein NJ, Albers GW (2012). Comparison of arterial spin labeling and bolus perfusion-weighted imaging for detecting mismatch in acute stroke. Stroke.

[82] Hendrikse J, Petersen ET, Chèze A (2009). Relation between cerebral perfusion territories and location of cerebral infarcts. Stroke.

[83] Chalela JA, Alsop DC, Gonzalez-Atavales JB (2000). Magnetic resonance perfusion imaging in acute ischemic stroke using continuous arterial spin labeling. Stroke.

[84] Fernández-Seara MA, Edlow BL, Hoang A (2008). Minimizing acquisition time of arterial spin labeling at 3T. Magn Reson Med.

[85] Chen T-Y, Chiu L, Wu T-C (2012). Arterial spin-labeling in routine clinical practice: a preliminary experience of 200 cases and correlation with MRI and clinical findings. Clin Imaging.

[86] Chen J, Licht DJ, Smith SE (2009). Arterial spin labeling perfusion MRI in pediatric arterial ischemic stroke: initial experiences. J Magn Reson Imaging.

[87] Wintermark P, Warfield SK (2012). New insights in perinatal arterial ischemic stroke by assessing brain perfusion. Trans Stroke Res.

[88] Pienaar R, Paldino MJ, Madan N (2012). A quantitative method for correlating observations of decreased apparent diffusion coefficient with elevated cerebral blood perfusion in newborns presenting cerebral ischemic insults. Neuroimage.

[89] Njemanze PC, Beck OJ, Gomez CR (1991). North american symptomatic carotid endarterectomy trial. Methods, patient characteristics, and progress. Stroke.

[90] European Carotid Surgery Trialists’ Collaborative Group (1998). Randomised trial of endarterectomy for recently symptomatic carotid stenosis: final results of the MRC European Carotid Surgery Trial (ECST). Lancet.

[91] Stoll M, Hamann GF (2002). Cerebrovascular reserve capacity. Nervenarzt.

[92] Bokkers RPH, Osch MJPV, Klijn CJM (2011). Cerebrovascular reactivity within perfusion territories in patients with an internal carotid artery occlusion. J Neurol, Neurosurg Psychiatry.

[93] Siero JCW, Hartkamp NS, Donahue MJ (2015). Neuronal activation induced BOLD and CBF responses upon acetazolamide administration in patients with steno-occlusive artery disease. Neuroimage.

[94] Van Laar PJ, Hendrikse J, Mali WPTM (2007). Altered flow territories after carotid stenting and carotid endarterectomy. J Vasc Surg.

[95] Hesselink JR (2006). Differential diagnostic approach to MR imaging of white matter diseases. Top Magn Reson Imaging.

[96] Zhang Q, Stafford RB, Wang Z (2012). Microvascular perfusion based on arterial spin labeled perfusion MRI as a measure of vascular risk in Alzheimer’s disease. J Alzheimers Dis.

[97] Bastos-Leite AJ, Kuijer JPA, Rombouts SARB (2008). Cerebral blood flow by using pulsed arterial spin-labeling in elderly subjects with white matter hyperintensities. AJNR Am J Neuroradiol.

[98] van Osch MJP, Teeuwisse WM, van Walderveen MAA (2009). Can arterial spin labeling detect white matter perfusion signal?. Magn Reson Med.

[99] Mutsaerts HJMM, Richard E, Heijtel DFR (2013). Gray matter contamination in arterial spin labeling white matter perfusion measurements in patients with dementia. Neuroimage Clin.

[100] Wang DJJ, Alger JR, Qiao JX (2013). Multi-delay multi-parametric arterial spin-labeled perfusion MRI in acute ischemic stroke—comparison with dynamic susceptibility contrast enhanced perfusion imaging. Neuroimage Clin.

[101] Alsop DC, Detre JA (1996). Reduced transit-time sensitivity in noninvasive magnetic resonance imaging of human cerebral blood flow. J Cereb Blood Flow Metab.

[102] Yoo R-E, Yun TJ, Rhim JH (2015). Bright vessel appearance on arterial spin labeling MRI for localizing arterial occlusion in acute ischemic stroke. Stroke.

[103] Wang J, Alsop DC, Song HK (2003). Arterial transit time imaging with flow encoding arterial spin tagging (FEAST). Magn Reson Med.

[104] Zaharchuk G, Bammer R, Straka M (2009). Arterial spin-label imaging in patients with normal bolus perfusion-weighted MR imaging findings: pilot identification of the borderzone sign. Radiology.

[105] Chng SM, Petersen ET, Zimine I (2008). Territorial arterial spin labeling in the assessment of collateral circulation: comparison with digital subtraction angiography. Stroke.

[106] Huang Y-C, Liu H-L, Lee J-D (2013). Comparison of arterial spin labeling and dynamic susceptibility contrast perfusion MRI in patients with acute stroke. PLoS One.

[107] Wolf RL, Wang J, Detre JA (2008). Arteriovenous shunt visualization in arteriovenous malformations with arterial spin-labeling MR imaging. AJNR Am J Neuroradiol.

[108] Brayne C, Davis D (2012). Making Alzheimer’s and dementia research fit for populations. Lancet.

[109] McMahon PM, Araki SS, Sandberg EA (2003). Cost-effectiveness of PET in the diagnosis of Alzheimer disease. Radiology.

[110] Donahue MJ, Lu H, Jones CK (2006). An account of the discrepancy between MRI and PET cerebral blood flow measures. A high-field MRI investigation. NMR Biomed.

[111] Xu G, Rowley HA, Wu G (2010). Reliability and precision of pseudo-continuous arterial spin labeling perfusion MRI on 3.0 T and comparison with 15o-water PET in elderly subjects at risk for Alzheimer’s disease. NMR Biomed.

[112] Wolk DA, Detre JA (2012). Arterial spin labeling MRI: an emerging biomarker for Alzheimer’s disease and other neurodegenerative conditions. Curr Opin Neurol.

[113] Chen Y, Wolk DA, Reddin JS (2011). Voxel-level comparison of arterial spin-labeled perfusion MRI and FDG-PET in Alzheimer disease. Neurology.

[114] Musiek ES, Chen Y, Korczykowski M (2012). Direct comparison of fluorodeoxyglucose positron emission tomography and arterial spin labeling magnetic resonance imaging in Alzheimer’s disease. Alzheimers Dement.

[115] Austin BP, Nair VA, Meier TB (2011). Effects of hypoperfusion in Alzheimer’s disease. J Alzheimers Dis.

[116] Alexopoulos P, Sorg C, Förschler A (2012). Perfusion abnormalities in mild cognitive impairment and mild dementia in Alzheimer’s disease measured by pulsed arterial spin labeling MRI. Eur Arch Psychiatry Clin Neurosci.

[117] Yoshiura T, Hiwatashi A, Noguchi T (2009). Arterial spin labelling at 3-T MR imaging for detection of individuals with Alzheimer’s disease. Eur Radiol.

[118] Mak HKF, Chan Q, Zhang Z (2012). Quantitative assessment of cerebral hemodynamic parameters by QUASAR arterial spin labeling in Alzheimer’s disease and cognitively normal elderly adults at 3-tesla. J Alzheimers Dis.

[119] Hu WT, Wang Z, Lee VM-Y (2010). Distinct cerebral perfusion patterns in FTLD and AD. Neurology.

[120] Du AT, Jahng GH, Hayasaka S (2006). Hypoperfusion in frontotemporal dementia and Alzheimer disease by arterial spin labeling MRI. Neurology.

[121] Steketee RME, Bron EE, Meijboom R, et al. (2015) Early-stage differentiation between presenile Alzheimer’s disease and frontotemporal dementia using arterial spin labeling MRI. Eur Radiol 10.1007/s00330-015-3789-x PMC4666273 26024845

[122] Chao LL, Buckley ST, Kornak J (2010). ASL perfusion MRI predicts cognitive decline and conversion from MCI to dementia. Alzheimer Dis Assoc Disord.

[123] Kim SM, Kim MJ, Rhee HY (2013). Regional cerebral perfusion in patients with Alzheimer’s disease and mild cognitive impairment: effect of APOE epsilon4 allele. Neuroradiology.

[124] Dashjamts T, Yoshiura T, Hiwatashi A (2011). Simultaneous arterial spin labeling cerebral blood flow and morphological assessments for detection of Alzheimer’s disease. Acad Radiol.

[125] Bron EE, Steketee RME, Houston GC (2014). Diagnostic classification of arterial spin labeling and structural MRI in presenile early stage dementia. Hum Brain Mapp.

[126] Zou J-X, Wang M-J, Lei X-J, Chen X-G (2014). 3.0T MRI arterial spin labeling and magnetic resonance spectroscopy technology in the application of Alzheimer’s disease. Exp Gerontol.

[127] Wang Z (2014). Characterizing early Alzheimer’s disease and disease progression using hippocampal volume and arterial spin labeling perfusion MRI. J Alzheimers Dis.

[128] Schuff N, Matsumoto S, Kmiecik J (2009). Cerebral blood flow in ischemic vascular dementia and Alzheimer’s disease, measured by arterial spin-labeling magnetic resonance imaging. Alzheimers Dement.

[129] Yoshiura T, Hiwatashi A, Yamashita K (2009). Simultaneous measurement of arterial transit time, arterial blood volume, and cerebral blood flow using arterial spin-labeling in patients with Alzheimer disease. AJNR Am J Neuroradiol.

[130] Mazza M, Marano G, Traversi G (2011). Primary cerebral blood flow deficiency and Alzheimer’s disease: shadows and lights. J Alzheimers Dis.

[131] Raji CA, Lee C, Lopez OL (2010). Initial experience in using continuous arterial spin-labeled MR imaging for early detection of Alzheimer disease. AJNR Am J Neuroradiol.

[132] Wierenga CE, Hays CC, Zlatar ZZ (2014). Cerebral blood flow measured by arterial spin labeling MRI as a preclinical marker of Alzheimer’s disease. J Alzheimers Dis.

[133] Wang Z, Das SR, Xie SX (2013). Arterial spin labeled MRI in prodromal Alzheimer’s disease: a multi-site study. NeuroImage: Clin.

[134] Alsop DC, Detre JA, Grossman M (2000). Assessment of cerebral blood flow in Alzheimer’s disease by spin-labeled magnetic resonance imaging. Ann Neurol.

[135] Johnson NA, Jahng G-H, Weiner MW (2005). Pattern of cerebral hypoperfusion in Alzheimer disease and mild cognitive impairment measured with arterial spin-labeling MR imaging: initial experience. Radiology.

[136] Alsop DC, Casement M, de Bazelaire C (2008). Hippocampal hyperperfusion in Alzheimer’s disease. Neuroimage.

[137] Asllani I, Habeck C, Scarmeas N (2008). Multivariate and univariate analysis of continuous arterial spin labeling perfusion MRI in Alzheimer’s disease. J Cereb Blood Flow Metab.

[138] Ances BM, Liang CL, Leontiev O (2009). Effects of aging on cerebral blood flow, oxygen metabolism, and blood oxygenation level dependent responses to visual stimulation. Hum Brain Mapp.

[139] Dai W, Lopez OL, Carmichael OT (2009). Mild cognitive impairment and Alzheimer disease: patterns of altered cerebral blood flow at MR imaging. Radiology.

[140] Binnewijzend MAA, Kuijer JPA, Benedictus MR (2013). Cerebral blood flow measured with 3D pseudocontinuous arterial spin-labeling MR imaging in Alzheimer disease and mild cognitive impairment: a marker for disease severity. Radiology.

[141] Pfefferbaum A, Chanraud S, Pitel A-L (2010). Volumetric cerebral perfusion imaging in healthy adults: regional distribution, laterality, and repeatability of pulsed continuous arterial spin labeling (PCASL). Psychiatry Res.

[142] Lüdemann L, Warmuth C, Plotkin M (2009). Brain tumor perfusion: comparison of dynamic contrast enhanced magnetic resonance imaging using T1, T2, and T2* contrast, pulsed arterial spin labeling, and H2(15)O positron emission tomography. Eur J Radiol.

[143] Lehmann P, Monet P, de Marco G (2010). A comparative study of perfusion measurement in brain tumours at 3 tesla MR: arterial spin labeling versus dynamic susceptibility contrast-enhanced MRI. Eur Neurol.

[144] Järnum H, Steffensen EG, Knutsson L (2010). Perfusion MRI of brain tumours: a comparative study of pseudo-continuous arterial spin labelling and dynamic susceptibility contrast imaging. Neuroradiology.

[145] Hirai T, Kitajima M, Nakamura H (2011). Quantitative blood flow measurements in gliomas using arterial spin-labeling at 3T: intermodality agreement and inter- and intraobserver reproducibility study. AJNR Am J Neuroradiol.

[146] van Westen D, Petersen ET, Wirestam R (2011). Correlation between arterial blood volume obtained by arterial spin labelling and cerebral blood volume in intracranial tumours. MAGMA.

[147] Yamashita K, Yoshiura T, Hiwatashi A (2013). Differentiating primary CNS lymphoma from glioblastoma multiforme: assessment using arterial spin labeling, diffusion-weighted imaging, and (18)f-fluorodeoxyglucose positron emission tomography. Neuroradiology.

[148] Noguchi T, Yoshiura T, Hiwatashi A (2008). Perfusion imaging of brain tumors using arterial spin-labeling: correlation with histopathologic vascular density. AJNR Am J Neuroradiol.

[149] Yamashita K, Yoshiura T, Hiwatashi A (2012). Arterial spin labeling of hemangioblastoma: differentiation from metastatic brain tumors based on quantitative blood flow measurement. Neuroradiology.

[150] Nabavizadeh SA, Assadsangabi R, Hajmomenian M (2015). High accuracy of arterial spin labeling perfusion imaging in differentiation of pilomyxoid from pilocytic astrocytoma. Neuroradiology.

[151] Wolf RL, Wang J, Wang S (2005). Grading of CNS neoplasms using continuous arterial spin labeled perfusion MR imaging at 3 tesla. J Magn Reson Imaging.

[152] Chawla S, Wang S, Wolf RL (2007). Arterial spin-labeling and MR spectroscopy in the differentiation of gliomas. AJNR Am J Neuroradiol.

[153] Warmuth C, Gunther M, Zimmer C (2003). Quantification of blood flow in brain tumors: comparison of arterial spin labeling and dynamic susceptibility-weighted contrast-enhanced MR imaging. Radiology.

[154] Weber MA, Zoubaa S, Schlieter M (2006). Diagnostic performance of spectroscopic and perfusion MRI for distinction of brain tumors. Neurology.

[155] Kim HS, Kim SY (2007). A prospective study on the added value of pulsed arterial spin-labeling and apparent diffusion coefficients in the grading of gliomas. AJNR Am J Neuroradiol.

[156] Furtner J, Schöpf V, Schewzow K, et al. (2013) Arterial spin-labeling assessment of normalized vascular intratumoral signal intensity as a predictor of histologic grade of astrocytic neoplasms. AJNR Am J Neuroradiol 10.3174/ajnr.A3705 PMC7964716 23945226

[157] Cebeci H, Aydin O, Ozturk-Isik E (2014). Assessment of perfusion in glial tumors with arterial spin labeling; comparison with dynamic susceptibility contrast method. Eur J Radiol.

[158] Fellah S, Girard N, Chinot O (2011). Early evaluation of tumoral response to antiangiogenic therapy by arterial spin labeling perfusion magnetic resonance imaging and susceptibility weighted imaging in a patient with recurrent glioblastoma receiving bevacizumab. J Clin Oncol.

[159] Ozsunar Y, Mullins ME, Kwong K (2010). Glioma recurrence versus radiation necrosis? A pilot comparison of arterial spin-labeled, dynamic susceptibility contrast enhanced MRI, and FDG-PET imaging. Acad Radiol.

[160] Deibler AR, Pollock JM, Kraft RA (2008). Arterial spin-labeling in routine clinical practice, part 1: technique and artifacts. AJNR Am J Neuroradiol.

[161] Clement P, Mutsaerts H-J, Ghariq E et al (2014) Review of confounding effects on perfusion measurements. Front Hum Neurosci 8

[162] Takeuchi H, Taki Y, Hashizume H (2011). Cerebral blood flow during rest associates with general intelligence and creativity. PLoS One.

[163] O’Gorman RL, Kumari V, Williams SCR (2006). Personality factors correlate with regional cerebral perfusion. Neuroimage.

[164] Ainslie PN, Cotter JD, George KP (2008). Elevation in cerebral blood flow velocity with aerobic fitness throughout healthy human ageing. J Physiol.

[165] Chen JJ, Rosas HD, Salat DH (2011). Age-associated reductions in cerebral blood flow are independent from regional atrophy. Neuroimage.

[166] Paradiso S, Robinson RG, Boles Ponto LL (2003). Regional cerebral blood flow changes during visually induced subjective sadness in healthy elderly persons. J Neuropsychiatry Clin Neurosci.

[167] Mardimae A, Balaban DY, Machina MA (2012). The interaction of carbon dioxide and hypoxia in the control of cerebral blood flow. Pflugers Arch.

[168] Page KA, Chan O, Arora J (2013). Effects of fructose vs glucose on regional cerebral blood flow in brain regions involved with appetite and reward pathways. JAMA.

[169] Wang J, Rao H, Wetmore GS (2005). Perfusion functional MRI reveals cerebral blood flow pattern under psychological stress. Proc Natl Acad Sci U S A.

[170] Addicott MA, Yang LL, Peiffer AM (2009). The effect of daily caffeine use on cerebral blood flow: how much caffeine can we tolerate?. Hum Brain Mapp.

[171] Domino EF, Ni L, Xu Y (2004). Regional cerebral blood flow and plasma nicotine after smoking tobacco cigarettes. Prog Neuropsychopharmacol Biol Psychiatry.

[172] Jain V, Duda J, Avants B (2012). Longitudinal reproducibility and accuracy of pseudo-continuous arterial spin-labeled perfusion MR imaging in typically developing children. Radiology.

[173] Goff DA, Buckley EM, Durduran T (2010). Noninvasive cerebral perfusion imaging in high-risk neonates. Semin Perinatol.

[174] Gevers S, Nederveen AJ, Fijnvandraat K (2012). Arterial spin labeling measurement of cerebral perfusion in children with sickle cell disease. J Magn Reson Imaging.

[175] Dahmoush HM, Vossough A, Roberts TPL (2012). Pediatric high-field magnetic resonance imaging. Neuroimaging Clin N Am.

[176] Herscovitch P, Raichle ME (1985). What is the correct value for the brain–blood partition coefficient for water?. J Cereb Blood Flow Metab.

[177] Lu H, Clingman C, Golay X, van Zijl PCM (2004). Determining the longitudinal relaxation time (T1) of blood at 3.0 tesla. Magn Reson Med.

[178] Lu H, Golay X, Pekar JJ, Van Zijl PCM (2003). Functional magnetic resonance imaging based on changes in vascular space occupancy. Magn Reson Med.

[179] Dai W, Garcia D, de Bazelaire C, Alsop DC (2008). Continuous flow-driven inversion for arterial spin labeling using pulsed radio frequency and gradient fields. Magn Reson Med.

[180] Wong EC, Buxton RB, Frank LR (1998). A theoretical and experimental comparison of continuous and pulsed arterial spin labeling techniques for quantitative perfusion imaging. Magn Reson Med.

[181] Asllani I, Borogovac A, Brown TR (2008). Regression algorithm correcting for partial volume effects in arterial spin labeling MRI. Magn Reson Med.

[182] Melzer TR, Watts R, MacAskill MR (2011). Arterial spin labelling reveals an abnormal cerebral perfusion pattern in Parkinson’s disease. Brain.

[183] Fernández-Seara MA, Mengual E, Vidorreta M (2012). Cortical hypoperfusion in Parkinson’s disease assessed using arterial spin labeled perfusion MRI. Neuroimage.

[184] Wolf RC, Grön G, Sambataro F (2011). Magnetic resonance perfusion imaging of resting-state cerebral blood flow in preclinical Huntington’s disease. J Cereb Blood Flow Metab.

[185] Chen JJ, Salat DH, Rosas HD (2012). Complex relationships between cerebral blood flow and brain atrophy in early Huntington’s disease. Neuroimage.

[186] Ota M, Sato N, Nakata Y (2013). Abnormalities of cerebral blood flow in multiple sclerosis: a pseudocontinuous arterial spin labeling MRI study. Magn Reson Imaging.

[187] Paling D, Thade Petersen E, Tozer DJ (2014). Cerebral arterial bolus arrival time is prolonged in multiple sclerosis and associated with disability. J Cereb Blood Flow Metab.

[188] Marshall O, Lu H, Brisset J-C (2014). Impaired cerebrovascular reactivity in multiple sclerosis. JAMA Neurol.

[189] Golay X, Petersen ET (2006). Arterial spin labeling: benefits and pitfalls of high magnetic field. Neuroimaging Clin N Am.

[190] van Gelderen P, de Zwart JA, Duyn JH (2008). Pittfalls of MRI measurement of white matter perfusion based on arterial spin labeling. Magn Reson Med.

[191] Zuo Z, Wang R, Zhuo Y (2013). Turbo-FLASH based arterial spin labeled perfusion MRI at 7 T. PLoS One.

[192] Petersen ET, Lim T, Golay X (2006). Model-free arterial spin labeling quantification approach for perfusion MRI. Magn Reson Med.

[193] Qiu D, Straka M, Zun Z (2012). CBF measurements using multidelay pseudocontinuous and velocity-selective arterial spin labeling in patients with long arterial transit delays: comparison with xenon CT CBF. J Magn Reson Imaging.

[194] Duncan JS (2010). Imaging in the surgical treatment of epilepsy. Nat Rev Neurol.

[195] Holmes GL (2002). Seizure-induced neuronal injury: animal data. Neurology.

[196] Duncan JS (2002). Seizure-induced neuronal injury: human data. Neurology.

[197] Choi J, Koh S (2008). Role of brain inflammation in epileptogenesis. Yonsei Med J.

[198] Wolf RL, Alsop DC, Levy-Reis I (2001). Detection of mesial temporal lobe hypoperfusion in patients with temporal lobe epilepsy by use of arterial spin labeled perfusion MR imaging. AJNR Am J Neuroradiol.

[199] Liu HL, Kochunov P, Hou J (2001). Perfusion-weighted imaging of interictal hypoperfusion in temporal lobe epilepsy using FAIR-HASTE: comparison with H(2)(15)O PET measurements. Magn Reson Med.

[200] Lim Y-M, Cho Y-W, Shamim S (2008). Usefulness of pulsed arterial spin labeling MR imaging in mesial temporal lobe epilepsy. Epilepsy Res.

[201] Pizzini F, Farace P, Zanoni T (2008). Pulsed-arterial-spin-labeling perfusion 3T MRI following single seizure: a first case report study. Epilepsy Res.

[202] Pollock JM, Deibler AR, West TG (2008). Arterial spin-labeled magnetic resonance imaging in hyperperfused seizure focus: a case report. J Comput Assist Tomogr.

[203] Pendse N, Wissmeyer M, Altrichter S (2010). Interictal arterial spin-labeling MRI perfusion in intractable epilepsy. J Neuroradiol.

[204] Wissmeyer M, Altrichter S, Pereira VM (2010). Arterial spin-labeling MRI perfusion in tuberous sclerosis: correlation with PET. J Neuroradiol.

[205] Storti SF, Boscolo Galazzo I, Del Felice A (2014). Combining ESI, ASL and PET for quantitative assessment of drug-resistant focal epilepsy. Neuroimage.

[206] Pizzini FB, Farace P, Manganotti P (2013). Cerebral perfusion alterations in epileptic patients during peri-ictal and post-ictal phase: PASL vs DSC-MRI. Magn Reson Imaging.

[207] Toledo M, Munuera J, Salas-Puig X (2011). Localisation value of ictal arterial spin-labelled sequences in partial seizures. Epileptic Disord.

[208] Oishi M, Ishida G, Morii K (2012). Ictal focal hyperperfusion demonstrated by arterial spin-labeling perfusion MRI in partial epilepsy status. Neuroradiology.

[209] Kanazawa Y, Morioka T, Arakawa S (2015). Nonconvulsive partial status epilepticus mimicking recurrent infarction revealed by diffusion-weighted and arterial spin labeling perfusion magnetic resonance images. J Stroke Cerebrovasc Dis.

[210] Van Paesschen W (2004). Ictal SPECT. Epilepsia.

[211] Madan N, Grant PE (2009). New directions in clinical imaging of cortical dysplasias. Epilepsia.

[212] Théberge J (2008). Perfusion magnetic resonance imaging in psychiatry. Top Magn Reson Imaging.

[213] Duhameau B, Ferré J-C, Jannin P (2010). Chronic and treatment-resistant depression: a study using arterial spin labeling perfusion MRI at 3 tesla. Psychiatry Res.

[214] Ho TC, Wu J, Shin DD (2013). Altered cerebral perfusion in executive, affective, and motor networks during adolescent depression. J Am Acad Child Adolesc Psychiatry.

[215] Colloby SJ, Firbank MJ, He J (2012). Regional cerebral blood flow in late-life depression: arterial spin labelling magnetic resonance study. Br J Psychiatry.

[216] Scheef L, Manka C, Daamen M (2010). Resting-state perfusion in nonmedicated schizophrenic patients: a continuous arterial spin-labeling 3.0-T MR study. Radiology.

[217] Pinkham A, Loughead J, Ruparel K (2011). Resting quantitative cerebral blood flow in schizophrenia measured by pulsed arterial spin labeling perfusion MRI. Psychiatry Res.

[218] Kindler J, Jann K, Homan P (2015). Static and dynamic characteristics of cerebral blood flow during the resting state in schizophrenia. Schizophr Bull.

[219] Wolf RC, Thomann PA, Sambataro F (2012). Orbitofrontal cortex and impulsivity in borderline personality disorder: an MRI study of baseline brain perfusion. Eur Arch Psychiatry Clin Neurosci.

[220] Weiduschat N, Dubin MJ (2013). Prefrontal cortical blood flow predicts response of depression to RTMS. J Affect Disord.

[221] Homan P, Kindler J, Hauf M (2012). Cerebral blood flow identifies responders to transcranial magnetic stimulation in auditory verbal hallucinations. Transl Psychiatry.

[222] Handley R, Zelaya FO, Reinders AATS (2013). Acute effects of single-dose aripiprazole and haloperidol on resting cerebral blood flow (RCBF) in the human brain. Hum Brain Mapp.

[223] Nordin LE, Li T-Q, Brogren J (2013). Cortical responses to amphetamine exposure studied by PCASL MRI and pharmacokinetic/pharmacodynamic dose modeling. Neuroimage.

[224] Khalili-Mahani N, Niesters M, van Osch MJ (2015). Ketamine interactions with biomarkers of stress: a randomized placebo-controlled repeated measures resting-state fMRI and PCASL pilot study in healthy men. Neuroimage.

[225] Hendrikse J, van der Grond J, Lu H (2004). Flow territory mapping of the cerebral arteries with regional perfusion MRI. Stroke.

[226] Hartkamp NS, Helle M, Chappell MA (2014). Validation of planning-free vessel-encoded pseudo-continuous arterial spin labeling MR imaging as territorial-ASL strategy by comparison to super-selective P-CASL MRI. Magn Reson Med.

[227] Hartkamp NS, Petersen ET, De Vis JB (2013). Mapping of cerebral perfusion territories using territorial arterial spin labeling: techniques and clinical application. NMR Biomed.

